# Lipid-associated macrophages’ promotion of fibrosis resolution during MASH regression requires TREM2

**DOI:** 10.1073/pnas.2405746121

**Published:** 2024-08-22

**Authors:** Souradipta Ganguly, Sara Brin Rosenthal, Kei Ishizuka, Ty D. Troutman, Theresa V. Rohm, Naser Khader, German Aleman-Muench, Yasuyo Sano, Sebastiano Archilei, Pejman Soroosh, Jerrold M. Olefsky, Ariel E. Feldstein, Tatiana Kisseleva, Rohit Loomba, Christopher K. Glass, David A. Brenner, Debanjan Dhar

**Affiliations:** ^a^Department of Medicine, School of Medicine, University of California, San Diego, CA 92093; ^b^Cancer Genome and Epigenetics Program, Sanford Burnham Prebys Medical Discovery Institute, La Jolla, CA 92037; ^c^Center for Computational Biology and Bioinformatics, Department of Medicine, University of California, San Diego, CA 92093; ^d^Department of Cellular and Molecular Medicine, University of California, San Diego, CA 92093; ^e^Division of Allergy and Immunology, Department of Pediatrics, Cincinnati Children’s Hospital Medical Center, University of Cincinnati College of Medicine, Cincinnati, OH 45229; ^f^Cardiovascular and Metabolism discovery, Immunometabolism, Janssen Research & Development, La Jolla, CA 92121; ^g^Department of Pediatrics, School of Medicine, University of California, San Diego, CA 92093; ^h^Department of Surgery, School of Medicine, University of California, San Diego, CA 92093

**Keywords:** steatohepatitis, macrophage, fibrosis, Trem2, lipid associated macrophages (LAM)

## Abstract

While macrophage heterogeneity during metabolic dysfunction-associated steatohepatitis (MASH) has been described, the fate of these macrophages during MASH regression is poorly understood. Comparing macrophage heterogeneity during MASH progression vs regression, we identified specific macrophage subpopulations that are critical for MASH/fibrosis resolution. We elucidated the restorative pathways and gene signatures that define regression-associated macrophages and establish the importance of TREM2^+^ macrophages during MASH regression. Liver-resident Kupffer cells are lost during MASH and are replaced by four distinct monocyte-derived macrophage subpopulations. *Trem2* is expressed in two macrophage subpopulations: i) monocyte-derived macrophages occupying the Kupffer cell niche (MoKC) and ii) lipid-associated macrophages (LAM). In regression livers, no new transcriptionally distinct macrophage subpopulation emerged. However, the relative macrophage composition changed during regression compared to MASH. While MoKC was the major macrophage subpopulation during MASH, they decreased during regression. LAM was the dominant macrophage subtype during MASH regression and maintained *Trem2* expression. Both MoKC and LAM were enriched in disease-resolving pathways. Absence of TREM2 restricted the emergence of LAMs and formation of hepatic crown-like structures. TREM2^+^ macrophages are functionally important not only for restricting MASH-fibrosis progression but also for effective regression of inflammation and fibrosis. TREM2^+^ macrophages are superior collagen degraders. Lack of TREM2^+^ macrophages also prevented elimination of hepatic steatosis and inactivation of HSC during regression, indicating their significance in metabolic coordination with other cell types in the liver. TREM2 imparts this protective effect through multifactorial mechanisms, including improved phagocytosis, lipid handling, and collagen degradation.

## Significance

Since metabolic dysfunction-associated steatohepatitis (MASH) is the most common liver disease in the United States, the mechanisms underlying its regression are key to developing therapies. We elucidate the macrophage heterogeneity in livers undergoing MASH regression and demonstrate that lipid-associated macrophages (LAM) that emerge during MASH progression become the dominant macrophage subpopulation during regression and drive the restorative phenotype. LAMs express TREM2, and TREM2 is required for both the emergence of LAMs and their reparative functions. We propose efficient collagen degradation as a key protective mechanism mediated by TREM2 signaling. Absence of TREM2^+^ macrophages not only diminishes collagen resorption but also disrupts the metabolic coordination with other cell-types, leading to ineffective hepatic stellate cell inactivation and elimination of hepatic steatosis during regression.

Metabolic dysfunction-associated steatohepatitis (MASH), formerly known as nonalcoholic steatohepatitis (NASH), is a major risk factor for cirrhosis and hepatocellular carcinoma (HCC) and the second leading cause of liver transplants in the United States. Activation and recruitment of macrophages are an early response to hepatocyte injury that play an important role in MASH with fibrosis development. Although the molecular pathways in macrophages that promote MASH and fibrosis have been investigated, little is known about the pathways that restrain macrophage activation within the disease microenvironment.

Triggering receptors expressed on myeloid cells (TREM) are cell surface receptors that participate in diverse cellular processes (1, 2). MASH is associated with the emergence of lipid-associated macrophages (LAM) that highly express *Trem2* along with *Cd9, Gpnmb,* and *Spp1* (3–5) and are equivalent to the scar-associated macrophages (SAM) in human MASH (6–8). The functional role of hepatic LAM/SAMs, whether they promote (6, 7, 9) or protect (10, 11) against MASH, remains unresolved. LAMs/SAMs increase with MASH severity (3, 10), localize around steatotic hepatocytes (LAM) (3, 9, 10) and fibrotic scars (SAM) (6), activate hepatic stellate cells (HSC), the major fibrogenic cells in the liver, and promote TGF-β1-induced collagen deposition (7), suggesting that these TREM2^hi^ macrophages may be pathogenic. However, recent reports indicate that the TREM2 molecule itself plays a protective role in liver damage (12), steatosis (13), and MASH (14, 15).

Single-cell analyses have deepened our understanding of macrophage heterogeneity during MASH progression (3, 5, 6, 10, 16), but the composition of macrophage subpopulations during MASH regression remains obscure. For example, it is not known whether a distinct restorative macrophage subpopulation emerges during MASH regression. While *TREM2* expression increases in LAM/SAM during MASH progression, the fate and the functional role of these TREM2*^+^* macrophages during MASH regression are unknown. Furthermore, the identity and hallmarks of the key macrophage subpopulations that facilitate MASH and fibrosis resolution when the damage-evoking agent is removed (such as Western diet) are unknown.

Most of the existing knowledge regarding liver fibrosis resolution is derived from mouse models of chemical (CCl_4_ or acetaminophen)-induced fibrosis and regression (17–21). While these models offer insights into non-MASH-related liver fibrosis, they do not mimic the key pathophysiological aspects of human MASH patients such as obesity, steatosis, insulin resistance, global metabolic syndrome, and emergence of LAMs.

Here, we used two preclinical mouse models of MASH that resemble human pathobiology to elucidate macrophage heterogeneity in MASH progression and regression. We determine the dominant macrophage subtype during the various phases of the disease (healthy, MASH, and regression); identify the key macrophage subtype that facilitates MASH/fibrosis resolution; and address the mechanistic role of TREM2 in MASH/fibrosis resolution. Using single-cell RNAseq (scRNAseq), we identified four distinct macrophage clusters that expand during MASH and determined their fate during MASH regression. We further determined how the key cellular pathways enriched in these clusters are affected during regression. We demonstrate that the regression-associated macrophage (RAM) subpopulations that maintain LAM/SAM gene signatures, including high *Trem2* expression, are the key restorative macrophages that orchestrate MASH-fibrosis resolution. During MASH regression, absence of TREM2^+^ macrophages not only prevented efficient collagen resorption but also affected elimination of hepatic steatosis and HSC inactivation, indicating their significance in metabolic coordination with other cell types. Mechanistically, TREM2 regulates multiple restorative pathways, including increased collagenase activity, phagocytosis, improved lipid handling, thereby, conferring a protective (during MASH progression) as well as restorative roles (during resolution phases) to the LAM/SAMs.

## Methods

### Animal Experiments.

Mice with the *Alms1* mutation (*Foz/Foz* mice) were kindly provided by Dr. Geoffrey C. Farrell (Australian National University Medical School) and have been further characterized by our laboratory (22). *Trem2^−/−^* mice were from the Jackson Laboratory (Strain# 027197). *Foz::Trem2^−/−^* mice were generated by crossing *Foz/Foz* with *Trem2^−/−^* mice. All mice were on a C57BL/6J background, and littermates were used for experiments. Six- to eight-week-old (time point: 0 wk) males were placed on a Western diet (WD) (AIN-76A; Test Diet, St. Louis, MO, containing 40% fat, 15% protein, 44% carbohydrates based on caloric content, and 0.2% cholesterol) or on a standard chow diet (12% fat, 23% protein, and 65% carbohydrates) for up to 24 wk. For the regression study, Foz + WD and *Foz::Trem2^−/−^* + WD 12 wk mice were switched to the chow diet for an additional 8 to 12 wk. MASH was induced in WT and *Trem2^−/−^* males by feeding them WD along with 30% (w/v) fructose in drinking water (FrWD) for up to 24 wk. All animals were maintained in accordance with NIH guidelines and approved by the University of California San Diego Institutional Animal Care and Use Committee (IACUC, Protocol #S07022).

### Statistical Analysis.

Data are shown as means ± SEM. Group differences were compared using ANOVA and Student’s *t* test. Significance was set at *P* < 0.05 unless otherwise mentioned. GraphPad Prism was used for all nonsequencing analyses.

Additional methods are described in *SI Appendix*.

## Results

### TREM2 in MASH.

**Fig. 1.**
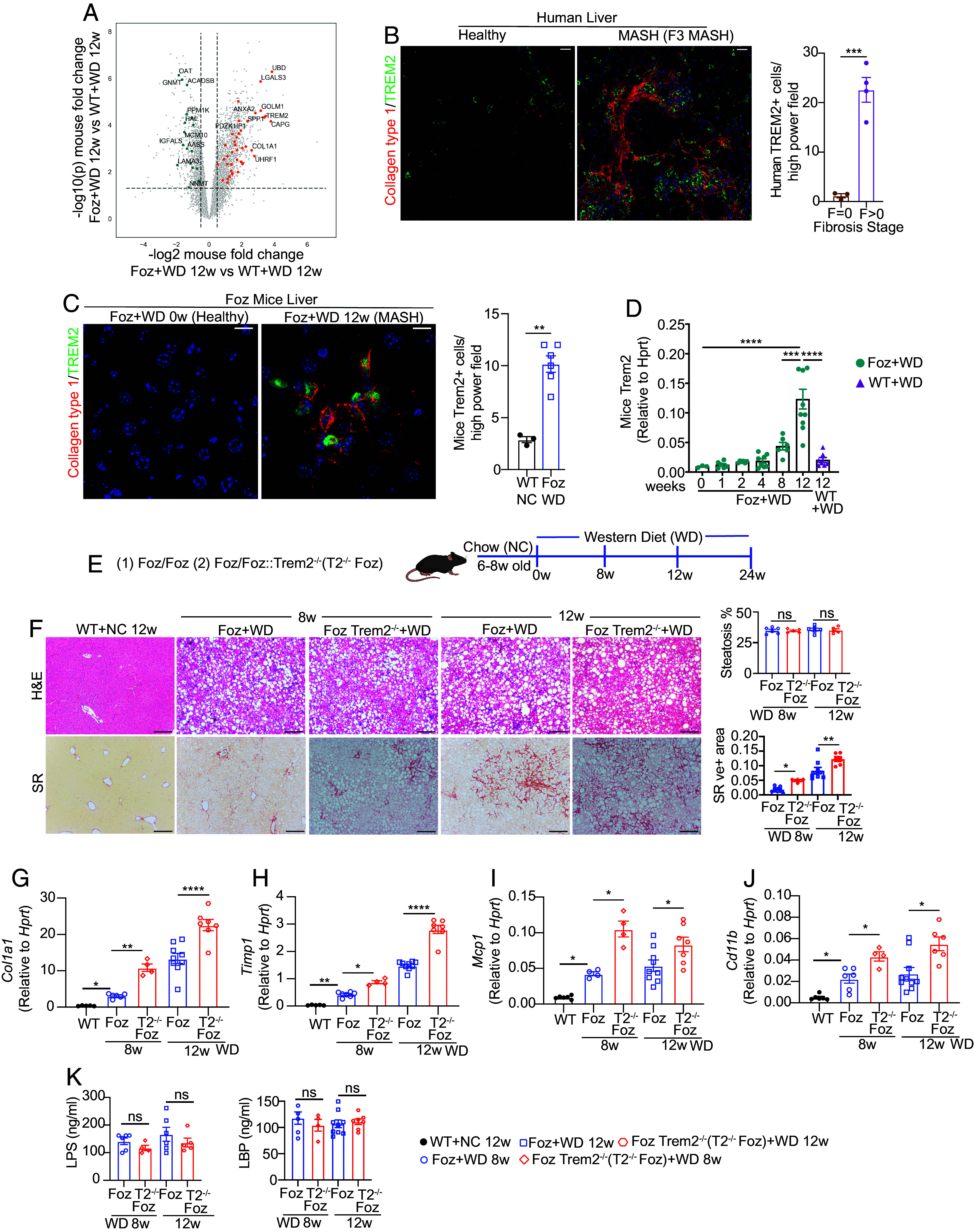
TREM2 in MASH. (*A*) Volcano plot of liver RNAseq demonstrating up-regulated (orange) and down-regulated (Green) genes common to mice and human MASH vs. healthy obese (see *SI Appendix* for methods). (*B* and *C*) Formalin-fixed paraffin-embedded (FFPE) human (*B*) and *Alms1^−/−^* (*Foz/Foz*) mice (*C*) liver sections were costained with anti-type 1 collagen (red) and TREM2 (green) antibodies (Scale bar, 30 µm for human and 10 µm for mouse sections) with corresponding quantifications. (*D*) qRT-PCR analysis of *Trem2* in WT and *Foz/Foz* livers. (*E*) Experimental design. Six- to eight-week-old *Foz/Foz* and *Foz::Trem2^−/−^* (T2^−/−^ Foz) mice were placed on WD for 8 to 24 wk. (*F*) Representative hematoxylin and eosin (H&E) and Sirius red (SR)–stained mouse liver sections and corresponding ImageJ quantifications. (Scale bar, 200 µm.) (*G–J*) Total liver RNA was subjected to qRT-PCR for (*G*) *Col1α1*, (*H*) *Timp1*, (*I*) *Mcp1*, and (*J*) *Cd11b*, gene expression. (*K*) Plasma lipopolysaccharide (LPS) and lipopolysaccharide-binding protein (LBP) levels were determined using ELISA. Data are expressed as mean ± SEM; one-way ANOVA and *T*-test; *P < 0.05, **P < 0.01, ***P < 0.001, ****P < 0.0001.

When fed a Western diet (WD), *Foz/Foz mice* (22) (Foz+WD) progress to MASH-fibrosis within 8 to 12 wk and to cirrhosis and HCC by 24 wk (22). The Foz+WD liver gene signature strongly correlates with that of human MASH (22). *TREM2* is one of the most highly up-regulated genes in both Foz and human MASH livers (Fig. 1*A*). Previous studies have reported that *TREM2* is expressed in recruited monocytes, liver-resident Kupffer cells, hepatic stellate cells (HSC), and endothelial cells (5, 12, 23). However, analyses of two publicly available scRNAseq datasets (3, 9) indicate that macrophages are the major cell types that express *TREM2* in the MASH livers (*SI Appendix*, Fig. S1.1*A*). In fibrotic human MASH as well as in *Foz/Foz* livers, TREM2^+^ macrophages are located next to collagen scars (Fig. 1 *B* and *C*). This spatial association has led to their SAM designation (6). TREM2^+^ macrophages are also classified as LAM that are found to be associated with hepatic crown-like structures (hCLS) surrounding hypertrophic lipid-laden hepatocytes (10) (*SI Appendix*, Fig. S1.1*B*) (discussed more in Fig. 5).

Although *TREM2* has been shown to be up-regulated in advanced MASH livers (6, 16), at what stage *TREM2* is induced during the disease progression and what event (such as steatosis, fibrosis, or hepatocyte death) triggers *TREM2* upregulation are not clear. A time-course analysis of *Trem2* expression in WT and *Foz/Foz* livers demonstrated the onset of *Trem2* upregulation in Foz + WD livers at 8 wk post-WD feeding (Fig. 1*D*), which coincides with the onset of liver injury (22). *Trem2* continued to increase as MASH and fibrosis progressed. *Trem2* expression remained low in WT + WD 12 wk mice, which only had MAFL (22) (Fig. 1*D*), indicating that *Trem2* expression is associated with hepatocyte death and liver inflammation during the progression of MAFL to MASH.

Recent reports indicate that TREM2 is protective during MASH development (14, 15). To model MASH regression and evaluate macrophage heterogeneity in livers undergoing MASH/fibrosis resolution as well as to evaluate the mechanistic role of TREM2^+^ macrophages in the various phases of the disease, we modeled MASH progression and regression using two different preclinical mouse models. To assess the functional role of TREM2 in the development of MASH-fibrosis in our models, we generated *Foz::Trem2^−/−^* mice, fed them WD, and compared them to Foz + WD mice (Fig. 1*E*). The absence of TREM2 did not affect body weight, liver weight (*SI Appendix*, Fig. S1.1*C*), liver injury (*SI Appendix*, Fig. S1.1*D*), or liver steatosis (Fig. 1*F*). However, *Foz::Trem2^−/−^* mice developed more fibrosis (Fig. 1 *F*–*H*) and inflammation (Fig. 1 *I* and *J*).

*Foz/Foz* mice advance to cirrhosis after 24 wk on WD^23^. At later time points (20 to 24 wk WD), the differences in fibrosis between the two groups diminished since the disease scores reached a point of saturation (*SI Appendix*, Fig. S1.1*E*). While the TREM2 slowed MASH development, the long-term continuation of WD-induced liver injury negated the TREM2-mediated protective mechanisms. This also indicates that TREM2 signaling axis alone is insufficient to provide complete protection against long-term MASH development. Therefore, when using *Foz/Foz* mice to study TREM2 signaling, we evaluated mice at 8 to 12 wk post-WD feeding.

Using a second model of MASH, we assessed whether *Trem2^−/−^* by itself (independent of the *Alms1* mutation found in *Foz/Foz* mice) produced the same phenotype (*SI Appendix*, Fig. S1.1*F*). When fed WD+30% fructose in drinking water (FrWD), both WT and *Trem2^−/−^* littermates had similar gains in body and liver weight liver injury and hepatic steatosis (*SI Appendix*, Fig. S1.1 *G*–*I*). However, deletion of *Trem2* resulted in more severe MASH and fibrosis (*SI Appendix*, Fig. S1.1 *I* and *J*), consistent with the *Foz/Foz* model.

Moreover, we found that *Trem2^−/−^* mice, but not their WT littermates, on FrWD progressed spontaneously from MASH-fibrosis to HCC (*SI Appendix*, Fig. S1.2 *A*–*D*), indicating that TREM2 is protective in MASH-associated HCC. The NT area showed extensive fibrosis (*SI Appendix*, Fig. S1.2*B*). About 60% of *Trem2^−/−^* mice developed an average of 5 tumors/liver with an average diameter of 2.5 mm (*SI Appendix*, Fig. S1.2*C*).

### Chemokines and Cytokines Affected by TREM2 Signaling.

Endotoxemia due to gut-barrier disruption and translocation of bacterial products (e.g., LPS) to the liver is a hallmark of MASH (22, 24). It remains unclear whether the aggravated hepatic inflammation in *Trem2^−/−^* mice is due to increased gut permeability. In both MASH models, the systemic levels of LPS and LPS binding protein (LBP) remained unaltered irrespective of the presence or absence of TREM2 (Fig. 1*K* and *SI Appendix*, Fig. S1.2*E*). Conversely, in the absence of TREM2, MASH livers had higher levels of activated Caspase 1 (*SI Appendix*, Fig. S1.2*F*), indicating that *Trem2^−/−^* MASH livers have enhanced NLRP3 inflammasome activation, a key activator of liver inflammation in MASH (25, 26). Indeed, consistent with our in vivo data, studies with WT and *Trem2^−/−^* BMDMs have reported an inherent hyperinflammatory phenotype of *Trem2^−/−^* BMDM in response to LPS (27) as well as an increased NLRP3 activation in the context of bacterial infection and neuro-inflammation (28–30).

Although *Trem2^−/−^*BMDMs have increased inflammatory responses (27), the specific hepatic chemokines/cytokines that are modulated by TREM2 signaling in MASH are unknown. We therefore designed a panel of 12 frequently studied cytokines/chemokines in MASH and subjected the liver lysates (from both preclinical models) to Luminex analyses. Both models had a significant increase in the levels of KC, MCP1, MIP2, IL1α, IL33, C1qR1, and S100A8 in the absence of TREM2, while the levels of TNFα, IL6, PDGFB, S100A9, and CRP were not significantly affected by TREM2 (*SI Appendix*, Fig. S1.2*G*).

### Absence of TREM2 Inhibits Resolution of MASH with Fibrosis.

MASH in Foz+WD 12 wk mice regresses when the WD is switched to a standard chow diet for an additional 8 to 12 wk (mimicking lifestyle modification in humans) (22). MASH regression is characterized by downregulation of profibrotic and inflammatory genes, HSC inactivation (31), and resolution of steatosis and fibrosis (22).

**Fig. 2.**
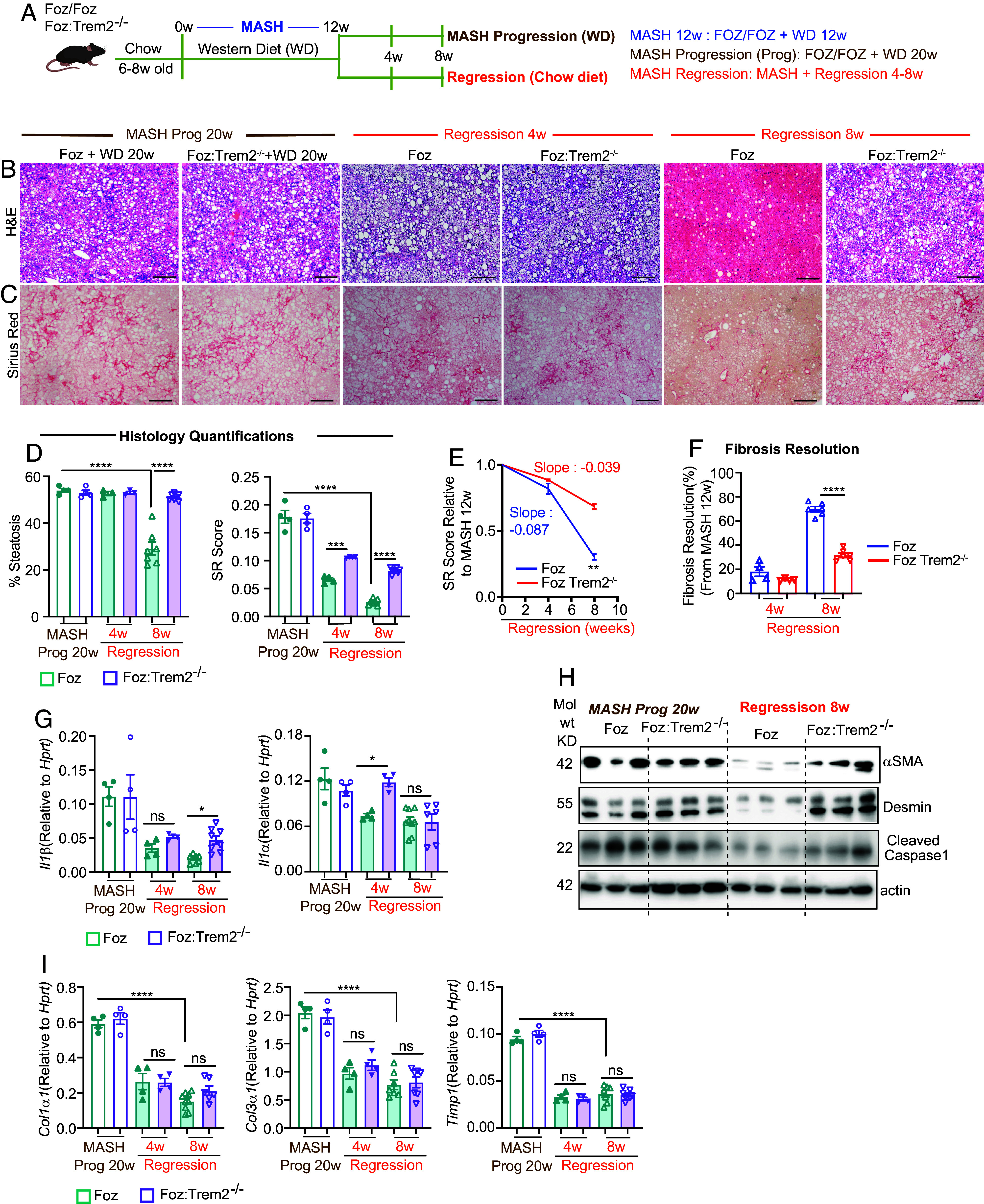
Absence of TREM2 prevents effective MASH and fibrosis resolution. (*A*) Experimental design: 6- to 8-wk-old *Foz/Foz* and *Foz::Trem2^−/−^* were fed WD for 12 wk to induce MASH/fibrosis. One cohort of mice were then switched to a chow diet for an additional 4 to 8 wk to model MASH regression. Another cohort continued WD as age-matched controls (MASH progression 20 wk). Three distinct time-points were analyzed: 1) MASH 12 wk (MASH-fibrosis immediately before regression), 2) MASH progression 20 wk (MASH-fibrosis in age-matched controls that continued WD), 3) MASH regression (4 and 8 wk regression). (*B* and *C*) Representative H&E (*B*) and SR-stained (*C*) mouse liver sections (Scale bar, 200 µm) and (*D*) ImageJ quantifications. (*E*) Linear regression analysis of SR quantification in *Foz/Foz* and *Foz::Trem2^−/−^* mice after 4 and 8 wk regression normalized to their respective 12 wk MASH SR scores. A greater negative slope in the *Foz/Foz* group indicates an increased rate of SR-reduction in these mice. (*F*) The extent of fibrosis resolution during MASH regression was quantified by normalizing SR-positive areas of *Foz/Foz* and *Foz::Trem2^−/−^* mice undergoing regression with the corresponding SR-positive areas at 12-wk MASH (refer to Fig. 1*F*). This was done to normalize for the higher SR scores of *Foz::Trem2^−/−^* + WD mice compared to Foz+WD mice at 12 wk, the time when the mice were put on regression. (*G*) Total liver RNA was analyzed by qRT-PCR for the expression of indicated inflammatory genes. (*H*) Liver lysates from regression and age-matched control mice were subjected to immunoblot analysis with the indicated antibodies. (*I*) Total liver RNA was analyzed by qRT-PCR for the expression of indicated genes. Data are expressed as mean ± SEM; one-way ANOVA; *P < 0.05, **P < 0.01, ***P < 0.001, ****P < 0.0001.

Although macrophages are pivotal in both the progression and regression of MASH (32), the characteristics of restorative macrophages that orchestrate fibrosis resolution during MASH regression have not been identified. To evaluate whether TREM2^+^ macrophages can modulate regression of MASH-fibrosis, we subjected Foz + WD 12 wk and *Foz::Trem2*^−/−^ + WD 12 wk MASH mice to a regression protocol by switching the WD back to a chow diet (22) (Fig. 2*A*). To model the kinetics of MASH/fibrosis resolution, we harvested tissues at 4 and 8 wk post initiation of regression. A separate cohort of age-matched mice continued WD for 8 wk (20 wk on WD total).

The WD to chow switch started to improve MASH within 4 wk with continued resolution through 8 wk. Strikingly, while *Foz/Foz* mice successfully eliminated >50% of hepatic fat deposits by 8 wk of WD to chow switch, *Foz::Trem2^−/−^* mice failed to eliminate hepatic steatosis (Fig. 2 *B* and *D*) and had higher liver weights compared to *Foz/Foz* regression mice (*SI Appendix*, Fig. S2*A*). Moreover, resolution of fibrosis in *Foz::Trem2^−/−^* mice was significantly impaired compared to the *Foz/Foz* mice (Fig. 2 *C*–*F*). *Foz/Foz* mice and *Foz::Trem2^−/−^* had equivalent steatosis and fibrosis when the mice continued on WD till 20 wk (Fig. 2 *C*–*F*), since the disease phenotype catches up by this time (as discussed in *SI Appendix*, Fig. S1.1*E*).

*Foz::Trem2^−/−^* + WD 12 wk mice had more fibrosis than *Foz/Foz* mice (Fig. 1*F*) when they were subjected to regression. Therefore, to evaluate the effective reduction in collagen levels following the dietary switch, the SR score of each regression group was normalized to the corresponding MASH SR score (at 12 wk WD just before the switch) as previously reported for CCl_4_ fibrosis resolution studies (33). The resulting linear regression plot (Fig. 2*E*) shows a significantly greater fibrosis reduction in *Foz/Foz* mice (slope: −0.087) compared to *Foz::Trem2^−/−^* (slope: −0.039). Similarly, percent fibrosis resolution (normalized to 12 wk MASH scores) also indicates significantly greater fibrosis reduction in *Foz/Foz* mice (70% resolution at 8 wk) compared to *Foz::Trem2^−/−^* mice (30% resolution at 8 wk) undergoing regression (Fig. 2*F*).

Absence of TREM2 slowed the resolution of liver inflammation, as indicated by higher residual *Il1α* and *Il1β* gene expression (Fig. 2*G**)* and higher NLRP3 activation (Cleaved Caspase 1 levels) (Fig. 2*H*). Absence of TREM2 also impaired effective HSC inactivation (31) indicated by higher residual levels of αSMA (activated HSC marker) and desmin (total HSC marker) in *Foz::Trem2^−/−^* regressed mice compared to their *Foz/Foz* counterparts (Fig. 2*H* and *SI Appendix*, Fig. S2*B*). Interestingly, even though *Foz::Trem2^−/−^* mice had greater residual liver collagen deposits, the gene expression of *Col1*α*1* and *Col3α1* were equally suppressed during regression in *Foz/Foz* and *Foz::Trem2^−/−^* mice (Fig. 2*I*). Therefore, the higher amount of residual collagen in *Foz::Trem2^−/−^* mice (Fig. 2 *D*−*F*) most likely reflects a defect in collagen degradation.

### Macrophage Heterogeneity during MASH Progression and Regression.

**Fig. 3.**
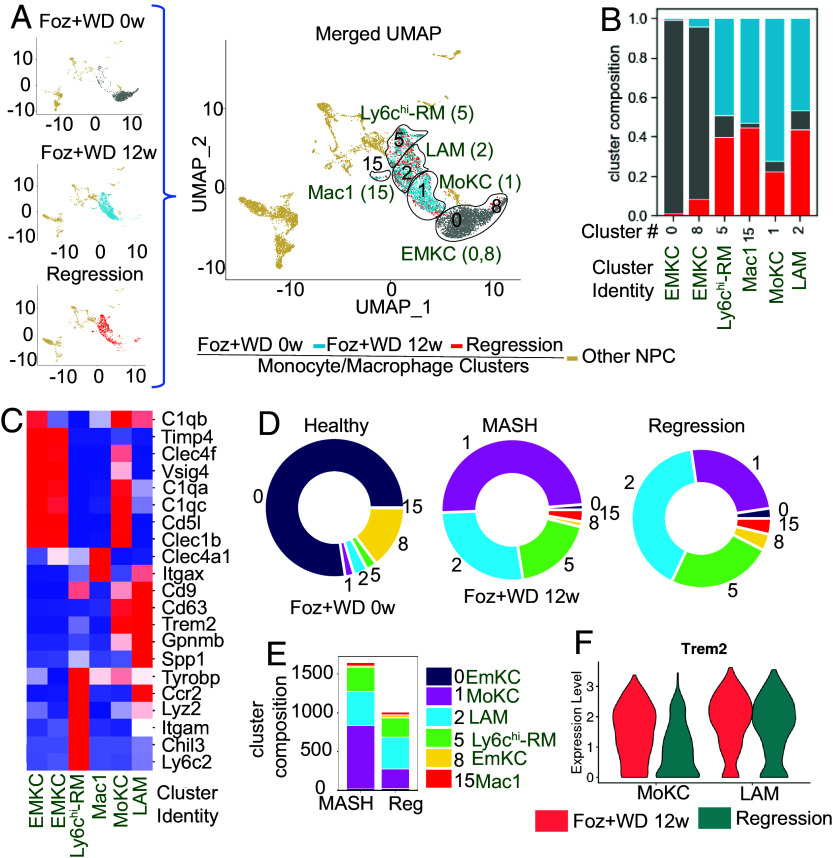
Macrophage heterogeneity during MASH progression and regression. (*A–F*) Total nonparenchymal cells (NPC) isolated from healthy (Foz + WD 0 wk), MASH (Foz + WD 12 wk), and MASH-regression (Foz + WD 12 wk + Chow 8 wk) mice were subjected to scRNAseq. This analysis focuses on cells of macrophage and monocyte origin. Other NPCs, such as T-cells, B-cells, and dendritic cells (listed in *SI Appendix*, Fig. S3*A*), were excluded from this analysis. (*A*) UMAPs highlighting Monocyte-macrophage clusters, including cells from healthy, MASH, and regression. Individual UMAPs from each condition are shown in the *Left* panel and the merged UMAP on the *Right.* (*B*) Cluster composition plot showing the relative proportion of cells from healthy, MASH, and regression across all the clusters. (*C*) Heatmap showing relative average expression of cell-type marker genes (*Y* axis) with their cluster identity (*X* axis) for monocyte-macrophage subclusters. (*D* and *E*) Cluster composition plot showing the relative makeup (*D*), and cell counts (*E*), of each cluster in healthy control (Foz + WD 0 wk) and during MASH (Foz + WD 12 wk) and regression. (*F*) Violin plot showing *Trem2* expression in the MoKC and LAM during MASH progression and regression.

We analyzed macrophage heterogeneity in Foz + WD 12 wk MASH livers and the fate of these MASH-associated macrophage subpopulations during MASH regression. We further elucidate the key molecular pathways by which TREM2 exerts its protective function. scRNAseq was performed on immune cells isolated from healthy (Foz + WD 0 wk), MASH (Foz + WD 12 wk), and regression mouse livers (*SI Appendix*, Fig. S3 *A* and *B*). From the 18 different immune cell clusters (*SI Appendix*, Fig. S3 *A* and *B* and Dataset S1), we focused our analysis on 6 monocyte/macrophage clusters (clusters 0, 1, 2, 5, and 15) (Fig. 3*A*). The identity of each macrophage subcluster was assigned based on previously published cell-type identity marker genes (3, 5, 6, 10, 16) (Fig. 3 *B* and *C* and *SI Appendix*, Fig. S3*C*).

As expected, chow→WD switch changed the macrophage transcriptome (Fig. 3 *A* and *B*). In contrast, WD→chow (regression) did not induce dramatic changes in macrophage heterogeneity, and no new subcluster emerged during MASH regression compared to MASH (Fig. 3 *A* and *B*). Regression macrophage subclusters (red) aligned with MASH macrophage subclusters (Blue) (Fig. 3 *A* and *B*).

Clusters 0 and 8 were represented by macrophages from the healthy livers and were enriched with *Timd4^+^*embryo-derived Kupffer cells (EmKCs) (clusters 0, 8: *Timd4^+^, Vsig4^+^, Clec1b^+^, Clec4f^+^, Ccr2^−^*) (Fig. 3 *A*−*C* and *SI Appendix*, Fig. S3*C*). In MASH livers the *Timd4^+^*EmKCs were depleted, as reported (3, 5, 10, 34), and were replaced by four distinct *Timd4^-^*clusters (clusters 5, 15, 1, 2) that were derived from circulating monocytes, also known as recruited macrophages (RM) (Fig. 3 *A*−*C* and *SI Appendix*, Fig. S3*C*).

Cluster 5 (hereafter referred as Ly6c^hi^ RM) had a characteristic gene expression of previously described (5) Ly6c-high macrophages that were *Ly6c2^hi^, Chil3^hi^, Ccr2^hi^, Lyz2^hi^, Fn1^hi^*, whereas cluster 15 (hereafter referred as Mac1) highly expressed *Itgax* (Cd11c) and *Clec4a1* along with *Fcrl5, Fabp4, Ighm, Cd300e, Fcgr4* (Fig. 3 *A*–*C* and *SI Appendix*, Fig. S3*C*). A defining feature of Cluster 2 was high *Spp1* (osteopontin) and *Gpnmb* expression (Fig. 3 *A*–*C* and *SI Appendix,* Fig. S3*C*), similar to the recently described hepatic LAM subpopulation (3). All three clusters (clusters 2, 5, and 15) lack expression of EmKC markers (*Vsig4, Clec4f, Clec1b,* and *C1q*). Cluster 1 highly expressed *Adgre1* (F4/80), along with several other EmKC markers *Vsig, Clec4f, Clec1b, C1q* even though they remain *Timd4^-^, Cd63^int^, Cd9^int^, Gpnmb^int^* (Fig. 3 *A*–*C* and *SI Appendix*, Fig. S3*C*). Thus, cluster 1 represents macrophages that are similar to but distinct from the EmKCs known as monocyte-derived Kupffer cells (MoKC) (3, 5, 10, 34), which occupy the EmKC niche during MASH.

*Trem2* is highly expressed in both LAM and MoKC subpopulations (Fig. 3*C*). Besides *Trem2*, both LAM and MoKC subpopulation express *Gpnmb*, *Cd9,* and *Fabp5* (albeit higher expression in LAM compared to MoKC) (*SI Appendix*, Fig. S3 *C–E*), that are characteristics of SAM described in humans (6, 7). This indicates that SAM might be a subset of LAM and/or MoKC.

### MoKC and LAM Are the Dominant Macrophage Subpopulations during MASH Progression and Regression Respectively.

We analyzed the scRNAseq data to evaluate the relative makeup of each macrophage cluster in healthy control (Foz + WD 0 wk), during MASH progression (Foz + WD 12 wk), and MASH regression (Fig. 3*D*). EmKCs (clusters 0 and 8), the major macrophage subtype in the healthy livers, are replaced by the four monocyte-derived macrophage populations [MoKC (1), LAM (2), Ly6chi-RM (5), and Mac1 (15)] during MASH (Fig. 3*D*). Among these, MoKC (Cluster 1) was the most dominant macrophage subpopulation (Fig. 3*D*). During MASH regression, the total macrophage population decreases, as expected, because of improvement in overall liver health due to the removal of the disease-triggering WD (Fig. 3 *D* and *E*). In regression livers, while no new transcriptionally distinct macrophage subpopulation emerged (compared to MASH), the relative macrophage cluster composition changed. During MASH regression, MoKC population shrunk (Fig. 3 *D* and *E*), while the LAM population (Cluster 2) remained constant (Fig. 3*E*), making it the major macrophage subpopulation during regression (Fig. 3 *D* and *E*). Although MoKC still expressed *Trem2* during regression, its expression was reduced compared to that in MASH (Fig. 3*F*). The LAMs, however, maintained high *Trem2* expression during both MASH and regression (Fig. 3*F*). EmKC populations (clusters 0 and 8) that were mostly lost during MASH did not return (albeit a slight recovery) during the 8 wk of regression (Fig. 3 *D* and *E*).

### TREM2 in MASH-Associated Macrophages Identifies Distinct Functional Pathways.

**Fig. 4.**
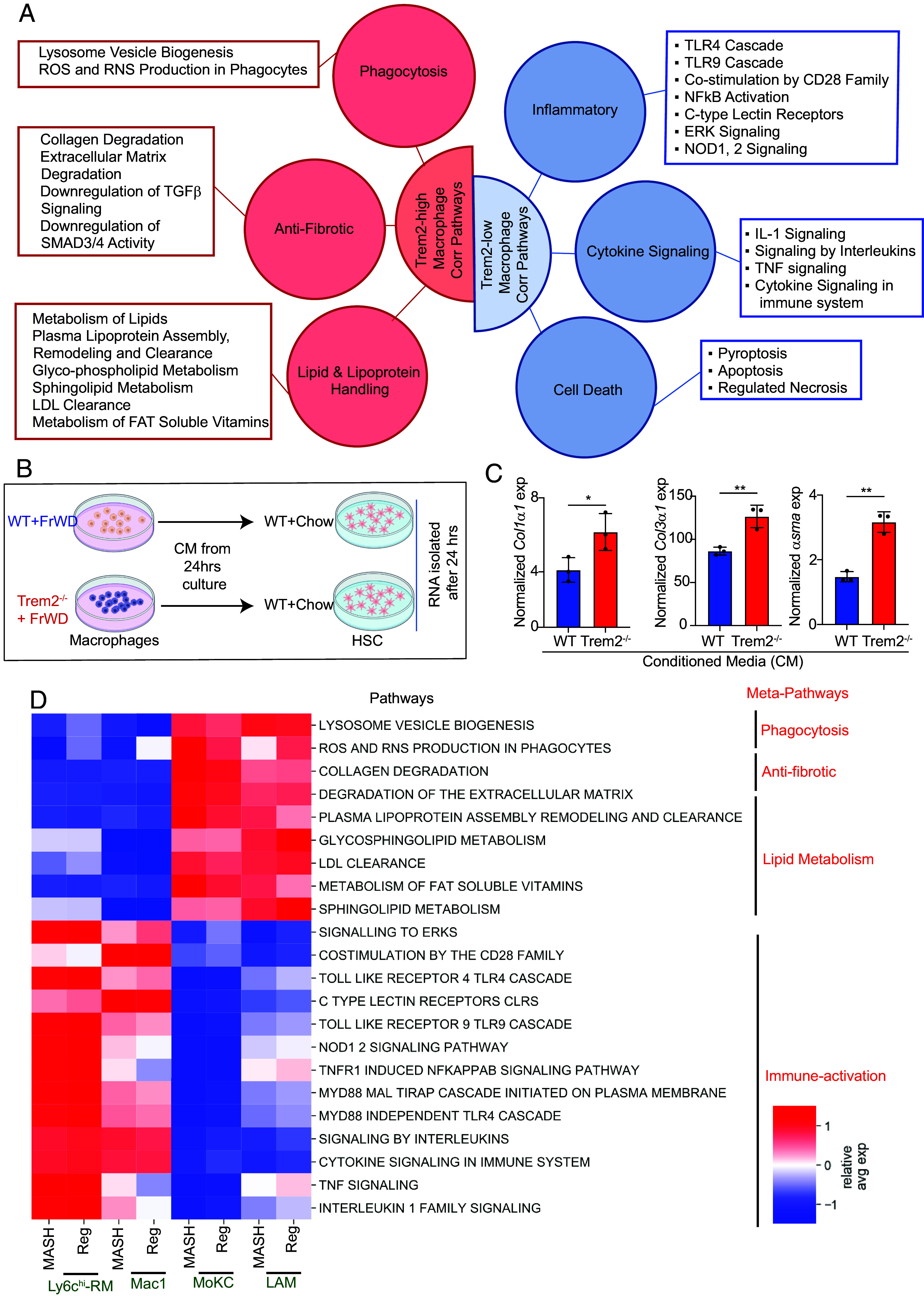
Pathways affected by TREM2 in MASH and regression-associated macrophages. (*A*) A cartoon summarizing the meta-pathways (red/blue circles) and the corresponding GSEA pathways (boxes) which are positively (red) or negatively (blue) correlated with *Trem2* expression in macrophages during MASH progression (see *SI Appendix* for methods). (*B*) Experimental design. Conditioned media (CM) from liver macrophages isolated from 24 wk FrWD-fed WT and *Trem2^−/−^* mice were added to HSC isolated from chow-fed WT mice. (*C*) Total RNA isolated from the HSC 24 h post CM incubation was subjected to qRT-PCR to identify gene expression changes in fibrogenic markers. Gene expression is normalized to HPRT as well as to the number of live macrophages that produced the corresponding CM. (*D*) Heatmap showing relative mean expression of the genes belonging to *Trem2* correlated pathways, of clusters 1, 2, 5, and 15 during MASH progression (MASH) and MASH regression (Reg) (see *SI Appendix* for methods). Data are expressed as mean ± SEM; *T*-test; *P < 0.05, **P < 0.01.

The macrophages in MASH livers are more inflammatory and profibrogenic compared to the macrophages residing in a healthy liver (*SI Appendix*, Fig. S4*A*). To identify the key biological differences among macrophage population that exist within the same MASH liver but are distinguished based on *Trem2* expression levels, we first computed the Pearson correlation of all genes with *Trem2* across all macrophage clusters (clusters 0, 1, 2, 5, 8, and 15). These correlation values were then used in a gene set enrichment analysis (GSEA) to identify pathways enriched with genes that are positively or negatively correlated with *Trem2* expression (Fig. 4*A*). We found that the majority of significantly enriched “Reactome pathways” (rectangular boxes, Fig. 4*A*) can be broadly categorized into six major meta-pathways (Red/Blue circles, Fig. 4*A*). Genes positively correlated with high *Trem2* expression in MASH-associated macrophages are enriched for meta-pathways such as phagocytosis, anti-fibrosis, and improved lipid/lipoprotein handling. On the other hand, MASH-associated macrophages expressing low levels of *Trem2* were enriched for meta-pathways that correspond to inflammation, enhanced cytokine signaling, and cell death (apoptosis, pyroptosis, and regulated necrosis) (Fig. 4*A* and Datasets S2 and S3).

Trem2^hi^ macrophages are enriched in antifibrotic pathways, involving ECM degradation, and downregulation of TGF-β and related fibrotic signaling. However, conditioned media from TREM2^+^ SAM were reported to be more fibrogenic compared to their TREM2^−^ non-scar-associated counterparts (6). We hypothesized that the absence of TREM2 in SAM would make them even more fibrogenic. To test this, we isolated macrophages from WT and *Trem2^−/−^* MASH mice and used the conditioned media to stimulate primary HSC isolated from healthy mice. Conditioned media from *Trem2^−/−^* MASH macrophages activated primary HSC more than the conditioned media from WT MASH-associated macrophages, as evidenced by increased *Col1α1*, *Col3α1,* and α*SMA* gene expression in HSC (Fig. 4 *B* and *C*). Thus, TREM2 dampens the fibrotic effect of macrophages within the MASH environment.

To elucidate the functional role of different macrophage subpopulations in MASH progression and how these functions are altered during regression, we performed pathway enrichment analysis on the differentially regulated genes within the macrophage subpopulations during MASH and compared them with MASH regression. Within the MASH macrophage populations, the MoKC and LAM were relatively less inflammatory and enriched in protective pathways during MASH progression (Fig. 4*D*), consistent with the observation that MoKC and LAMs highly express *Trem2* (Fig. 3*C*), and Trem2^hi^ macrophages positively correlate these protective pathways (Fig. 4*A*). During regression, both MoKC and LAM continued to highly express disease-resolving pathways such as efficient phagocytosis, superior lipid handling, ECM degradation, and anti-fibrotic pathways (Fig. 4*D*). On the other hand, Ly6c^hi^-RM and Mac1 continued to be enriched in inflammatory pathways even during the regression phase (Fig. 4*D*).

Differential gene expression analysis revealed that only 25 genes changed significantly during regression in the LAM (*SI Appendix*, Fig. S4 *B* and *C*) and 75 genes in MoKC (*SI Appendix*, Fig. S4 *B* and *D*), suggesting that regression did not induce a profound shift in the transcriptome signature of individual macrophage subclusters. Since new macrophage subpopulations emerge during MASH that are maintained during regression, it is not feasible to perform head-to-head cluster-based comparisons with healthy macrophages (which are primarily Kupffer cells that get depleted during MASH). Gene expression analyses of all macrophage subclusters in healthy, MASH, and regression groups indicate that most genes dysregulated during MASH progression do not return to baseline levels during regression (*SI Appendix*, Fig. S4*E*). We further dissected the status of these genes across various macrophage subtypes during MASH regression (thereby identifying the regression-specific macrophage clusters enriched with these genes). Even though the profibrotic/inflammatory gene signature remained high during regression, the profibrotic/inflammatory genes were primarily expressed in the Ly6g^hi^ macrophage subcluster (*SI Appendix*, Fig. S4 *E*, *Right* panel). On the other hand, the reparative genes (such as anti-fibrotic and phagocytic) were predominantly expressed in the MoKC and LAM subsets. Therefore, at 8 wk regression, the relative sizes of various macrophage subpopulations were altered more substantially (compared to MASH) than the gene expression. It is possible that the myeloid population needs longer time to return to homeostasis.

### Absence of TREM2 Suppresses Emergence of LAM Subpopulation during MASH Progression and Regression.

MASH is associated with the emergence of hepatic LAM. LAMs highly express *Gpnmb, Cd9,* and *Trem2* and form distinctive hCLS around the steatotic regions within the liver (*SI Appendix*, Fig. S1.1*B*) (10). The functional role of LAMs and hCLS in MASH is debated (10, 35). Even though the inflammation and fibrosis is exacerbated in *Trem2^−/−^* mice, the impact of *Trem2* on evolution and functionality of hepatic LAMs has not been evaluated.

**Fig. 5.**
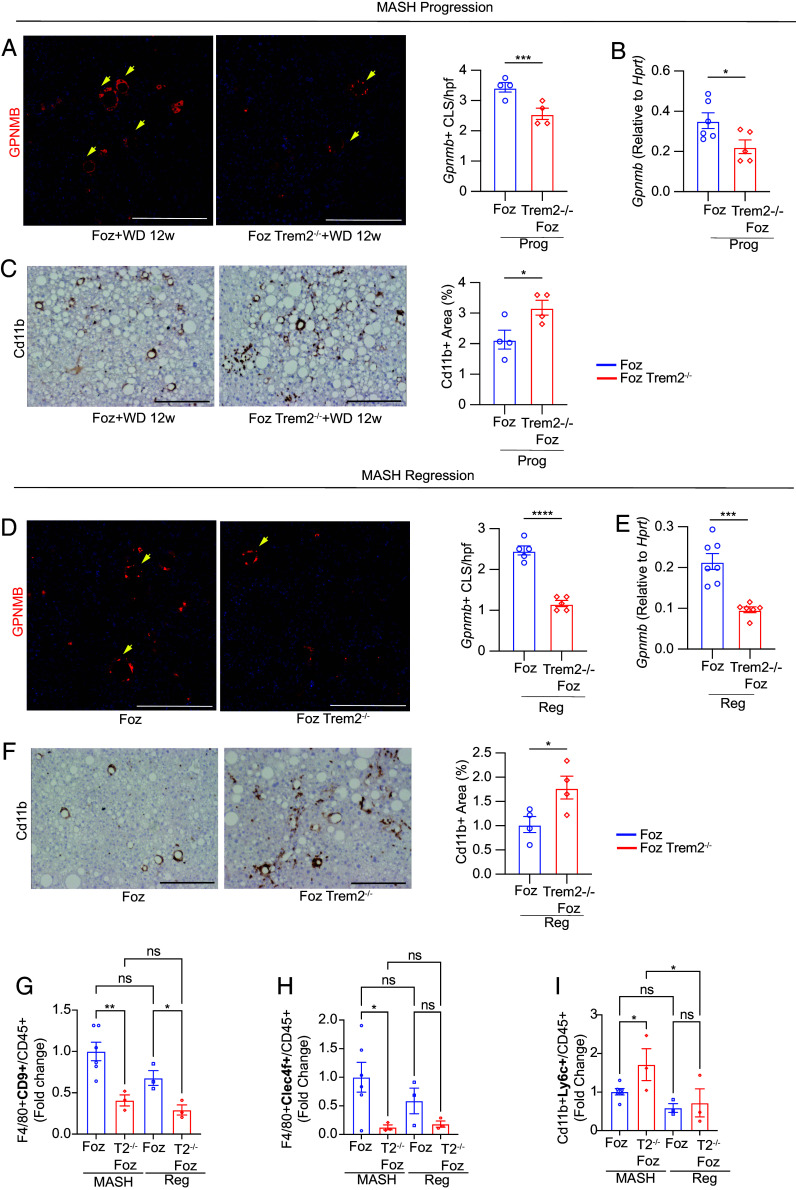
Absence of TREM2 suppresses emergence of LAMs during MASH progression and regression. (*A–C*) MASH progression. (*A*) Representative images of GPNMB (red) (DAPI = blue) (Scale bar, 200 µm) stained FFPE liver sections from *Foz/Foz* and *Foz::Trem ^−/−^* mice undergoing MASH progression (12 wk). Corresponding quantification of GPNMB^+^ hCLS in each randomly selected high-power field (hpf) on the *Right*. (*B*) qRT-PCR analysis of total liver RNA for *Gpnmb* expression. (*C*) FFPE sections from these mice were also stained with anti-Cd11b antibody. (Scale bar, 200 µm.) Representative images are shown with corresponding quantifications. (*D–F*) MASH regression. (*D*) Representative images of GPNMB (red) (DAPI = blue) (Scale bar, 200 µm) stained FFPE liver sections and quantification. (*E*) qRT-PCR analysis of total liver RNA for *Gpnmb* expression. (*F*) Representative Cd11b stained images of FFPE liver sections with quantification. (*G–I*) Flow cytometry of liver NPC isolated from *Foz/Foz* and *Foz::Trem2^−/−^* mice undergoing MASH progression and regression for LAM (CD9^+^), MoKC (Clec4f^+^), and Ly6C^hi^ macrophage subpopulations. Bar diagrams show the fold change in the proportion of (*G*) F4/80^+^CD9^+^, (*H*) F4/80^+^Clec4f^+^, and (*I*) Cd11b^+^Ly6c^+^ cells in the indicated groups compared to the Foz + WD 12 wk MASH group. Data are expressed as mean ± SEM; one-way ANOVA; *P < 0.05, **P < 0.01, ***P < 0.001, ****P < 0.0001.

Immunohistochemistry reveals that GPNMB^+^ LAMs form fewer hCLS in the absence of *Trem2* (Fig. 5*A* and *SI Appendix*, Fig. S5*A*). *Gpnmb* expression is also reduced in the absence of Trem2 (Fig. 5*B*). Intriguingly, despite increased inflammatory cell infiltration in *Trem2^−/−^* MASH livers (as indicated by abundance of Cd11b^+^ cells) (Fig. 5*C* and *SI Appendix*, Fig. S5*B*), there is a paradoxical decrease in GPNMB^+^ LAM in these mice (Fig. 5 *A* and *B* and *SI Appendix*, Fig. S5*A*).

GPNMB^+^ hCLS were also less abundant in the *Trem2^−/−^* regression mice (Fig. 5 *D* and *E*), despite having a significant residual lipid load (*Foz::Trem2^−/−^* regression vs. *Foz/Foz* 8 wk regression, Fig. 2 *B* and *D*) and more residual infiltrating macrophages (Fig. 5*F*). Overall, our data indicate that MASH progression and regression in the absence of TREM2 are associated with a lower number of LAMs/hCLS along with an increased residual collagen burden.

To further investigate how absence of TREM2 influences the overall macrophage heterogeneity during MASH progression and regression, we performed flow cytometry analyses of primary liver macrophages from *Foz/Foz* and *Foz::Trem2^−/−^* mice (Fig. 5 *G*–*I* and *SI Appendix*, Fig. S5 *C–K*). We observed that the abundance of Cd9^+^ (Fig. 5*G*) and Clec4f^+^ (Fig. 5*H*) macrophages (equivalent of LAM and MoKC, respectively, that have beneficial roles) was significantly lower in *Foz::Trem2^−/−^* livers compared to their WT counterparts in both MASH and regression. On the other hand, the inflammatory Ly6c^+^ macrophages (Fig. 5*I*) were more abundant in the absence of TREM2.

### LAMs Are the Major Restorative Macrophage Population during MASH Regression.

**Fig. 6.**
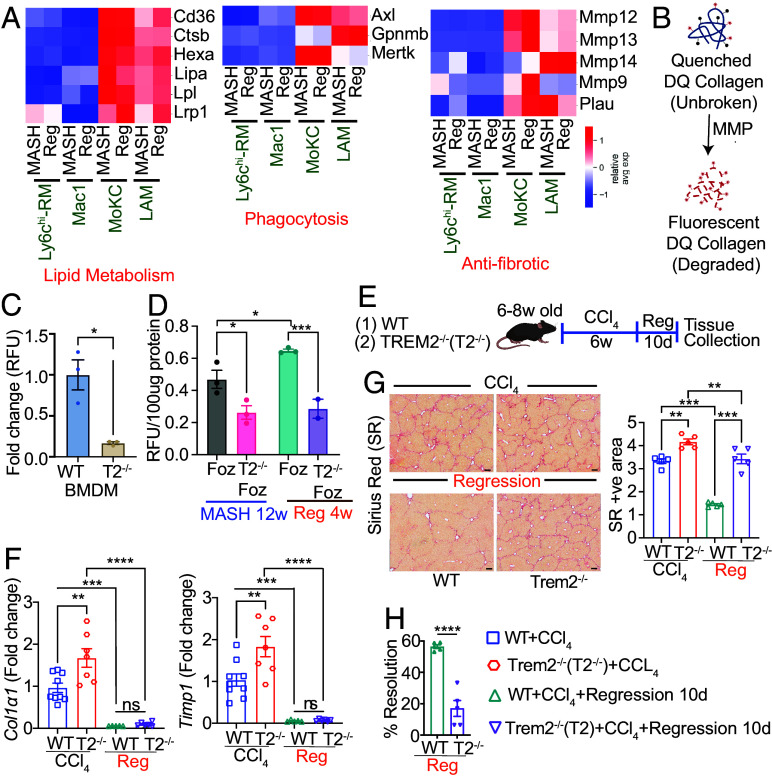
TREM2^+^ macrophages have high collagenolytic activities. (*A*) scRNAseq data from MASH and regression macrophage subpopulations (clusters 1, 2, 5, and 15) was analyzed as described in Figs. 3 and 4. Heat map showing relative expression of indicated genes belonging to reparative pathways (lipid handling, phagocytosis, and anti-fibrotic), among the various macrophage subpopulations during MASH and regression (Reg). These genes were selected based on a previous report (20), for their association with restorative macrophage phenotype during regression of CCl_4_-induced liver fibrosis. Only statistically significant genes (*P* < 0.05) were plotted. (*B–D*) Biochemical analysis of collagen degradation. (*B*) The fluorescent signal in intact fluorescein-labeled DQ^TM^ type-1 collagen remains quenched. Upon substrate hydrolysis by collagenases, the fluorescence signal increases which is used to measure enzymatic activity. (*C*) WT and *Trem2^−/−^* BMDMs were subjected to DQ^TM^ collagen assay. The measured fluorescence intensity was normalized to the cell count and represented as fold change compared to WT BMDM. (*D*) *Foz/Foz* and *Foz::Trem2^−/−^* mice were subjected to MASH (12 wk WD) and regression (4 wk). Macrophages isolated from the livers of indicated groups and were subjected to the DQ^TM^ collagen assay. The fluorescence intensity was normalized to the protein content of the cell lysates and plotted. (*E–H*) WT and *Trem2^−/−^* mice were administered CCl_4_ for 6 wk, followed by 10 d recovery. (*E*) Experimental design. (*F*) Total liver RNA was subjected to qRT-PCR for *Col1α1* and *Timp1* genes, normalized to *Hprt*, and plotted as fold change compared to the WT + CCl_4_ group. (*G*) Representative SR-stained mouse liver sections (Scale bar, 200 µm) and ImageJ quantifications. (*H*) The extent of fibrosis resolution during regression of CCl_4_-induced liver fibrosis was calculated by normalizing SR-positive areas of WT and *Trem2^−/−^* mice undergoing regression with the corresponding SR-positive areas at 6-wk CCl_4_ dosing. Data are expressed as mean ± SEM; one-way ANOVA; *P < 0.05, **P < 0.01, ***P < 0.001, ****P < 0.0001.

While the restorative macrophages that play key roles in fibrosis resolution have been studied in the context of chemical-induced fibrosis regression (17, 20), their identity during MASH resolution is unknown. During regression of CCl_4_-induced liver fibrosis, a new Ly6c^lo^ restorative macrophage population emerges that play a pivotal role in orchestrating fibrosis resolution, in part through the expression of matrix-degrading metalloproteinases (MMPs) (17, 20). We interrogated the gene signatures of CCl_4_ regression-associated restorative macrophages (20) in our MASH and regression livers. Both MoKC and LAM are Ly6c^lo^ macrophages (Fig. 3*C*), that emerge in MASH livers and express several of these gene signatures, including genes for ECM degradation (*Mmp9, Mmp12, Mmp13, Mmp14, Plau)*, lipid metabolism (*Cd36, Ctsb, Hexa, Lipa, Lpl, Lrp1*), and phagocytosis (*Axl, Gpnmb, Mertk*) (Fig. 6*A*). To identify additional markers of restorative macrophages, we evaluated the relative expression levels of all *Trem2*-correlated genes that are significantly altered among the various macrophage subpopulations during MASH vs regression. The resulting heat-map reveals several genes that are specifically up-regulated in the LAM and MoKC subpopulations (*SI Appendix*, Fig. S6 *A–D*, compare MASH vs. Reg for various macrophage subpopulations).

Interestingly, the expression of these restorative macrophage-associated genes is either sustained or further increased during MASH resolution (Fig. 6*A* and *SI Appendix*, Fig. S6 *A–D*). This imparts a disease-protective phenotype to the LAM and MoKCs even in livers undergoing active MASH that is further sustained as a reparative function during MASH regression when the dietary trigger is removed. Overall, our data suggest that TREM2^+^ LAM (along with MoKC) are the restorative macrophage populations that facilitate MASH regression.

To confirm whether the restorative gene signatures of the MASH-associated macrophages and RAMs rely on *Trem2* expression (irrespective of the disease dynamics), BMDMs from healthy WT and *Trem2^−/−^* mice were cultured under conditions mimicking tissue repair and analyzed using Nanostring nCounter (*SI Appendix*, Fig. S6*E*). Under both Il-4 (M2a) and Il10+TGFβ (M2c) stimulation (36), *Trem2*^−/−^ BMDMs exhibited decreased expression of most genes associated with the restorative macrophage signature.

### TREM2^+^ Macrophages Have High Collagenolytic Activities.

As discussed above (Fig. 2*I*), the collagen gene expression was equally down-regulated in both Foz and Foz::*Trem2^−/−^* mice during regression. However, the residual fibrosis post WD→chow was significantly higher in Foz::*Trem2^−/−^* compared to the Foz livers (Fig. 2*B*) suggesting a defect in collagen degradation. Indeed, TREM2^lo^ macrophages had relatively lower expression of collagenase genes (Figs. 4*A* and 6*A*). To investigate whether the presence of Trem2 can directly affect the collagenolytic activity of the macrophages, we performed collagenase assays with BMDMs isolated from WT and *Trem2^−/−^* mice (Fig. 6 *B* and *C*). Compared to WT, *Trem2^−/−^* BMDMs were less efficient in collagen degradation (Fig. 6*C*). Next, we evaluated whether the same defect exists in TREM2 deficient macrophages residing within MASH and regression livers. Macrophages isolated from *Foz/Foz* and *Foz*::*Trem2^−/−^* livers undergoing either active MASH (WD 12 wk) or regression (4 wk post WD to chow switch) were evaluated for their ability to degrade collagen (Fig. 6*D*). During both MASH progression and regression phases, TREM2^+^ cells had much higher collagenolytic activities compared to TREM2^−^ (Fig. 6*D*). Importantly, while the collagenolytic activities of TREM2^+^ cells increased significantly during MASH regression (compared to MASH), the collagen degradation abilities of TREM2^-^ cells remained low (Fig. 6*D*). Overall, our scRNAseq data together with in vivo and in vitro biochemical assays demonstrate that TREM2^+^ macrophages in both MoKC and LAM compartments restrict fibrosis development during MASH and facilitate fibrosis resorption during regression.

The role of TREM2 in the resorption of the fibrotic scars was also evaluated in a CCl_4_-induced fibrosis model (Fig. 6*E*). Similar to our findings in MASH, CCl_4_ regression was associated with efficient suppression of fibrogenic genes in both WT and *Trem2^−/−^* livers (Fig. 6*F*). However, the resorption of existing collagen was significantly attenuated in the absence of *Trem2* (Fig. 6 *G* and *H*), supporting our findings that TREM2^+^ macrophages are needed for efficient resolution of fibrosis.

## Discussion

Macrophages play a central role in orchestrating MASH progression and resolution (37). While macrophage heterogeneity during MASH progression is well established, the fate of the various MASH-associated macrophage populations during regression is not known. Moreover, the key RAM subpopulation that drives MASH-fibrosis resolution remains obscure. This study defined the RAM heterogeneity in mouse livers undergoing MASH regression and compared their gene signature to the macrophages residing in active MASH livers. Furthermore, we demonstrate that TREM2^+^ macrophages are required for efficient MASH regression and fibrosis resorption.

Macrophage heterogeneity in regression livers resembles the active MASH. While no new macrophage subpopulation emerged during MASH regression, the dynamics of macrophage subpopulation changed compared to active MASH. As reported (10, 34), we also observed that the Kupffer cells (Clusters 0 and 8) that constitute the major macrophage population in healthy livers were lost (more than 90%) and replaced by bone marrow–derived macrophages during MASH progression (Fig. 3). Two predominant TREM2^hi^ macrophage subpopulations (MoKC and LAM) and two TREM2^lo^ macrophage subpopulations (Ly6c^hi^-RM and Mac1) emerged within the active MASH livers from circulating monocytes. These monocyte-derived macrophage subpopulations continue to populate the livers undergoing MASH regression.

The Kupffer cell population that was lost during MASH progression did not return to normal levels in regressed livers. It remains unknown whether the original yolk-sac-derived Kupffer cells ever return to original numbers and the consequence of this change in repeated liver injuries. In contrast, MASH-associated activated HSCs return to an inactive state during regression, that closely resembles the quiescent HSCs but is distinct enough to cluster as a separate population (31).

While MoKC are the major macrophage subpopulation during MASH progression, they decrease during regression with a modest decrease in *Trem2* expression. However, despite a significant decrease in the hepatic lipid load during regression, the hepatic LAM population does not shrink. Rather, LAMs become the major macrophage subpopulation during regression and maintains *Trem2* expression. A similar phenomenon was observed in adipose tissue, where adipose tissue LAMs increased during obesity, persisted after weight loss, and continued to express *Trem2* (38). Although LAM/SAM have been widely described as a major macrophage population during MASH, their role in MASH pathology remains poorly understood. Our study provides evidence that TREM2^+^ LAMs and MoKCs restrain MASH and fibrosis development during active disease phases, and LAMs become the major restorative macrophage population aiding disease resolution during regression.

TREM2^+^ hepatic LAMs are transcriptionally similar to adipose tissue LAMs, emerge during MASH, and are recruited to hCLS^8^. The role of hepatic LAM and hCLS in MASH (10, 35) as well as adipose tissue LAM and CLS during obesity (4, 39) remains unresolved. It is important to understand whether the immune cells associated with CLS (in both adipose and hepatic tissues) are protective or harmful. *Ccr2^−/−^* mice, that lack hepatic LAMs, have exacerbated MASH (10). We demonstrate that absence of *Trem2* also reduced the emergence of LAM and hCLS, both during progression and regression, and this reduction is associated with poor disease outcome. Our data indicate that TREM2^+^ LAMs (the major macrophage subpopulation during regression, enriched in beneficial pathways) along with TREM2^+^ MoKCs play pivotal roles in MASH and fibrosis resolution. A recent study indicates that macrophage-derived Osteopontin (a hallmark of hepatic LAM) is protective in MASH (11). Importantly, while MASH regression led to equivalent suppression of collagen mRNA expression, the degradation of existing fibrotic scar is impaired in the absence of TREM2. The explanation is revealed in our RNAseq data as well as biochemical assays demonstrating that TREM2^+^ macrophages have higher anti-fibrotic gene expression as well as collagen degradation activity than TREM2^−^ macrophages.

Whether the absence of TREM2 influences whole-body glucose metabolism and obesity remains unclear (4, 40, 41). A recent study using the same *Trem2^−/−^* mouse strain (exon-2 deleted, Jax Strain# 027197) used in our studies revealed that absence of TREM2 does not cause whole-body metabolic abnormalities. While some studies reported increased body and liver weight in *Trem2^−/−^* mice on HFD or WD (4, 12, 40), the Winn et al. study (41) and ours did not observe such differences. Whether these observed differences are due to the way *Trem2^−/−^* mice were generated [exon 2 deletion (41) vs. exon 3 to 4 deletion (27)] or due to other lab-independent factors remains unknown. Importantly, however, myeloid-cell-specific deletion of *Trem2* (*Trem2^ΔMye^*, generated by crossing Trem2-Floxed mice with Lyz2-Cre) did not affect gain of liver and body weight or hepatic steatosis (similar to our observation) (15).

The absence of TREM2 inhibited the regression of hepatic steatosis as well as HSC inactivation. How the absence of TREM2 in macrophage modulates HSC inactivation as well as hepatic lipid metabolism during regression needs further investigation. A recent study indicates that the absence of TREM2 changes the exosome miRNA profile that alters the macrophage–hepatocyte metabolic coordination in MAFL and sepsis (13).

Macrophages in MASH livers, in general, are fibrogenic and inflammatory compared to healthy livers. However, scRNAseq indicated that despite residing in the same MASH livers, TREM2^hi^ macrophages were less inflammatory and more anti-fibrotic than TREM2^lo^ macrophages. TREM2^hi^ macrophages in both MASH and regression livers were enriched in protective pathways such as phagocytosis, ECM degradation, and superior lipid handling. TREM2^lo^ macrophages, on the other hand, expressed disease-worsening pathways, such as inflammatory and cell death pathways. Comparing individual macrophage subpopulations from MASH progression vs. regression reveals that the macrophage subpopulations that were enriched in beneficial pathways during regression were already present in active MASH livers. The WD to chow switch allowed these beneficial macrophage subpopulations to decrease hepatic inflammation, resorb the fibrotic scars, and restore liver homeostasis, and TREM2 is in the hub of controlling these beneficial pathways.

TREM2^+^ macrophages were shown to be efficient in efferocytosis and removal of damaged hepatocytes (15). We have elucidated additional mechanisms by which TREM2^+^ macrophages restrain MASH pathology, as well as facilitate MASH with fibrosis resolution triggered by diet. Our studies demonstrate that TREM2 attenuates inflammasome activation and tissue inflammation while promoting phagocytosis, ECM degradation, and lipid metabolism. Inflammasome activation is a hallmark of MASH livers (42), and we found that TREM2 restrains NLRP3 inflammasome activation. Absence of TREM2 not only leads to pronounced Caspase 1 cleavage and activation but the resolution of this pathway is also affected during MASH regression.

Thus, our studies indicate that TREM2 agonists might be beneficial for MASH/fibrosis therapy and might be used as an adjuvant to facilitate MASH and fibrosis regression in patients undergoing lifestyle modification, weight loss, or bariatric surgery.

## Supplementary Material

Appendix 01 (PDF)

Dataset S01 (XLSX)

Dataset S02 (XLSX)

Dataset S03 (XLSX)

## Data Availability

The sequences and metadata reported in this paper have been deposited in the Gene Expression Omnibus (GEO) database, https://www.ncbi.nlm.nih.gov/geo (Accession No. GSE261829) (43). All other study data are included in the manuscript and supporting information.
