# Supplementary material for: Lipid-associated macrophages’ promotion of fibrosis resolution during MASH regression requires TREM2
Source: Proc Natl Acad Sci U S A. 2024 Aug 22;121(35):e2405746121. doi: 10.1073/pnas.2405746121 (PMC11363294; doi:10.1073/pnas.2405746121)
Supplement: Supplementary file 1 — Appendix 01 (PDF) [file pnas.2405746121.sapp.pdf]

PNAS

Supporting Information for

Lipid Associated Macrophages' Promotion of Fibrosis Resolution during MASH regression requires TREM2

Souradipta Ganguly[a,b], Sara Brin Rosenthal[c], Kei Ishizuka[a], Ty D. Troutman[d,e], Theresa V. Rohm[a], Naser Khader[a], German Aleman-Muench[f], Yasuyo Sano[f], Sebastiano Archilei[a], Pejman Soroosh[f], Jerrold M. Olefsky[a], Ariel E. Feldstein[g,1], Tatiana Kisseleva[h], Rohit Loomba[a], Christopher K. Glass[d,2], David A. Brenner[a,b,2], and Debanjan Dhar[a,b,2]

[a]Department of Medicine, School of Medicine, University of California, San Diego, CA 92093
[b]Cancer Genome and Epigenetics Program, Sanford Burnham Prebys Medical Discovery Institute, La Jolla, CA 92037
[c]Center for Computational Biology and Bioinformatics, Department of Medicine, University of California, San Diego, CA 92093
[d]Department of Cellular and Molecular Medicine, University of California, San Diego, CA 92093
[e]Division of Allergy and Immunology, Department of Pediatrics, Cincinnati Children's Hospital Medical Center, University of Cincinnati College of Medicine, Cincinnati, OH 45229
[f]CVM discovery, Immunometabolism, Janssen Research & Development, La Jolla, CA 92121
[g]Department of Pediatrics, School of Medicine, University of California, San Diego, CA 92093
[h]Department of Surgery, School of Medicine, University of California, San Diego, CA 92093

[1] Present address: Global Drug Discovery Novo Nordisk, Copenhagen, Denmark 2300.

[2]To whom correspondence may be addressed. Email: cglass@health.ucsd.edu (CG), dbrenner@sbpdiscovery.org (DB), or ddhar@sbpdiscovery.org (DD).

This PDF file includes:

Supporting Materials and Methods
Figures S1 to S6
Tables S1 and S2
Legends for Datasets S1 to S3
SI References

Other supporting materials for this manuscript include the following:

Datasets S1 to S3

SI APPENDIX

Materials and Methods

Human Liver Samples

Human liver samples were kindly provided by Prof. Tatiana Kisseleva, University of California, San Diego (UCSD). Deidentified livers declined for transplantation are obtained via Lifesharing OPO and used in this study. The patient consent was obtained by www.lifesharing.org. This project (171883XX) has been reviewed by the Director of the UCSD HRPP, IRB Chair, or IRB Chair's designee and is certified as not qualifying as human subjects research according to the Code of Federal Regulations, Title 45, part 46 and UCSD Standard Operating Policies and Procedures, and therefore does not require IRB review. Livers were graded for steatosis, inflammation, and fibrosis by a pathologist using a double-blinded method (1).

CCl_4-induced liver fibrosis and regression

Fibrosis was induced in 6-8 weeks old male WT and Trem2$^{-/-}$ mice on C57BL/6 background by intraperitoneal injection of CCl_4 twice a week for 6 weeks (2 ml/kg; diluted in corn oil 1:4). The regression group (Reg) underwent a recovery period of 10 days following the final CCL_4 administration.

Quantitative Polymerase Chain Reaction (qRT-PCR)

Total RNA was isolated using Trizol (Invitrogen, Carlsbad, CA) followed by Rneasy column (Qiagen, Valencia, CA). The cDNAs were synthesized with MultiScribe™ Reverse Transcriptase (ThermoFischer Scientific, Waltham, MA) according to the manufacturer's instructions. qRT-PCR was performed using a QuantStudio 3 Real-Time PCR system (Applied Biosystems, Carlsbad, CA). The expression levels of genes were calculated and normalized to housekeeping gene *Hprt* using the ΔΔCT method (Invitrogen). Primer sequences are shown in Table S1.

Immunoblotting

Immunoblots (Western blots) were carried out on total protein extracts from liver tissues. Frozen tissues were homogenized in RIPA buffer containing protease and phosphatase inhibitors (ThermoFischer Scientific, Waltham, MA), followed by centrifugation at 13000rpm. Protein concentrations were determined by BCA assay (Bio-Rad, Hercules, CA). Equal amounts of protein lysates were separated by SDS-PAGE, transferred to polyvinyl difluoride membrane, and subjected to immunoblot analysis for the indicated primary antibodies (Table S2). Proteins were visualized using the ECL detection system (Pierce, Rockford, IL) with the appropriate secondary antibodies.

Histology

The left lateral lobe of mice livers was fixed in 10% neutral-buffered formalin for 48 hours, embedded in paraffin (Formalin-fixed paraffin-embedded [FFPE]), sectioned, and processed for hematoxylin and eosin (H&E), picrosirius red stain (SR), and immunostaining with the indicated antibodies (Table S2). All histology quantifications were performed by ImageJ software (NIH). The SR positive area was normalized to the non-steatosis area and plotted as a bar graph. For the linear regression analysis (Figure 2E) and to quantify fibrosis resolution (Figure 2F, 6H) in Foz/Foz and Foz::Trem2$^{-/-}$ mice during MASH regression, the regression SR scores calculated as above were adjusted to their respective MASH progression-SR scores (0w Regression) and plotted using GraphPad prism. This normalization allowed for a cleaner visualization of the changes in SR-deposits over the course of MASH regression for both mouse models. For quantification of Trem2-immunofluorescence images (Figure 1B, C), Trem2-positively stained cells in five randomly imaged nonoverlapping high-power fields in each sample were manually counted and plotted. For quantifications of GPNMB staining (Figure 5A, D and S5A), the number of GPNMB^{+} hepatic crown-like structures (hCLS) in five randomly imaged

nonoverlapping high-power fields in each sample, were manually counted and plotted. For Cd11b (Figure 5 C, F and S5B) and aSMA (Figure S2B) quantification the percentage of Cd11b and aSMA positive area was plotted.

Primary liver cell isolation (Perfusion method):

Primary liver cells were isolated as described before (2). Briefly, mice livers were perfused by cannulation through the inferior vena cava with 0.33mg/ml pronase (Roche, Indianapolis, IN), followed by 0.67mg/ml collagenase D (Roche, Indianapolis, IN), for 5-10 minutes before *ex vivo* digestion in a solution containing pronase, collagenase D and DNase1 (Roche, Indianapolis, IN) for 10min in a rotating incubator at 37^0C. The dissociated cells were strained through a 100μm mesh. The cell suspension was centrifuged at 50g for 1 minute to remove the hepatocytes, supernatant was collected and centrifuged at 800g for 7 minutes. Cell pellet washed with GBSS-B buffer at 700g for 7 minutes. Cells were finally isolated by Nycodenz gradient centrifugation at 2000g for 20 minutes. The Hepatic stellate cells (HSC) layer (upper layer) and the other non-parenchymal cells (NPC) layer (lower layer) were then collected and washed with GBSS-B at 800g for 7 minutes and processed as described below.

Hepatic Stellate Cells (HSC): For *in vitro* experiments, HSC were plated at a density of 400,000 cells per well in a 24 well plate in DMEM supplemented with 10% FBS and 1% Antibiotic-Antimycotic (AA). Following overnight culture, cells were washed with PBS and maintained in DMEM with 2% FBS, and 1% AA.

Hepatic Macrophages: The NPC layer was further processed for macrophage enrichment. The hepatic macrophages were enriched from the NPC fraction by their ability to readily attach and spread on tissue culture plates (in RPMI media supplemented with 10% FBS and 1% AA for 20min), and subsequently washing off the non-adherent cells(3-5).

To generate conditioned media (CM) from liver macrophages, the macrophage-enriched population was cultured in RPMI with 2% FBS, 1% AA for 24 hrs. Conditioned media (CM) was collected, centrifuged, and was either used immediately or stored at -80°C. Live macrophage cell count was recorded at the end of the experiment for normalization.

Stimulation of HSC with macrophage-conditioned media: 250µl of the macrophage CM generated as described above was added to the isolated HSC for 24 hours. HSCs were harvested for total RNA isolation and subsequent qRT-PCR of fibrotic genes. Fibrotic gene expression was normalized to *Hprt* as well as the live macrophage cell count of the CM.

Collagenase activity assay

Collagenase activity was measured in isolated macrophages using a fluorescein-labeled substrate, DQ collagen type I, from bovine skin (Molecular Probes, Eugene, OR). Briefly, the isolated cells were lysed in a reaction buffer (0.05 M Tris·HCl, 0.15 M NaCl, 5 mM CaCl2, 0.2 mM sodium azide, pH 7.6) supplemented with 0.1% TritonX and incubated with 25ug/ml DQ Collagen in microplate wells at room temperature for 30min-3hrs. Fluorescence was measured using λ excitation = 485 nm and λ emission = 535 nm and normalized to the total protein content of the cell lysates.

Single cell RNAseq (scRNAseq):

Total non-parenchymal cells (NPC) were isolated as described above. The total NPC fraction was primarily dominated by endothelial cells. To take a deeper look into the various TREM2+ and TREM2- macrophage sub-populations, in the context of MASH and MASH regression, we enriched the immune cell fraction from the total NPC population (from healthy, MASH and MASH regression Foz mice) by sorting (FACS) out the CD31+ endothelial cells and subjected the immune cell fraction to 10X Genomics 3' scRNA-seq

(Chromium Next GEM Single Cell 3′ GEM, Library & Gel Bead Kit v3.1). scRNA-Seq data was aligned with Cell Ranger version 3.0 and, each library was mapped to the mm10 genome. The Seurat package (version 3.0) was used for processing and merging raw count data, normalization, and clustering. Gene expression of each cell was normalized by total transcript counts and multiplied by 10,000. Cells of suspected low quality were removed, which had too few genes expressed (<500), which had high mitochondrial gene expression (>5%). Visualization was performed with Uniform Manifold Approximation and Projection (UMAP) dimensionality reduction and the Louvain algorithm with a resolution of 0.5 was used to cluster the cells.

Cell Identity: Cell identity of the 18 immune cell clusters was determined from Panglao datasets (Dataset S1). Monocyte macrophage sub-clusters were identified based on previously reported cell identity markers (Figure 3C and S3C).

Identification of Trem2 Correlated Pathways: First, the Pearson correlation of all genes with the Trem2-gene expression was computed within the entire MASH (and/or MASH regression) macrophage populations. All macrophage clusters (clusters 0, 1, 2, 5, 8, and 15) were included in the analysis. This vector of correlation values was then input to Gene Set Enrichment Analysis (GSEA) to identify pathways enriched in genes positively or negatively correlated ($p<0.05$) with *Trem2* expression(6).

Transcriptomic Analysis of Foz/Foz MASH and human MASH:

To determine the similarity in gene expression of Foz/Foz MASH to human MASH, the hepatic transcription profile of 12-week Foz+WD (MASH+Fibrosis) vs 12-week WT+WD (Steatosis only) was compared with a publicly available human MASH vs healthy obese gene expression dataset GSE48452 as before (7). Genes whose expressions are significantly altered between Foz+WD 12w (MASH) and WT+WD 12w (simple steatosis) are initially plotted. Genes that are also significantly upregulated and downregulated in the

human dataset GSE48452 comparing MASH to healthy obese are determined and highlighted in orange and green respectively. A total of 257 genes are significantly dysregulated in both human and Foz mice MASH. Of these, 234 genes are dysregulated in the same direction in both species, indicating these genes are consistently upregulated or downregulated in both mice and humans. Among these commonly dysregulated genes, the 20 with the highest absolute fold change are annotated. Dotted vertical lines indicate log fold change of 0.5 or -0.5. Dotted horizontal line indicates p=0.05.

Luminex

To identify differences in cytokine and chemokine profiles in NASH livers that develop in the presence or absence of TREM2, we subjected liver lysates of 16 weeks fructose+WD (FrWD) fed WT and Trem2$^{-/-}$ mice to luminex analysis. Liver tissues were homogenized in 1 mL of the lysis buffer tissue extraction reagent 1 (ThermoFischer Scientific, Waltham, MA) containing EDTA-free protease inhibitor cocktail (Roche, Indianapolis, IN). Liver lysates or cell culture supernatants were analyzed with the Luminex mouse cytokine/chemokine magnetic bead-custom made (R&Dsystems, Minneapolis, MN). Samples were read on a Luminex MAGPIX Instrument (Luminex, Austin, TX) and MFIs (Mean Fluorescent Intensity) were normalized to absolute values with standard curves generated using the best-fit feature in the Masterplex software (Hitachi Solutions, Irvine, CA). Data normalization was performed using total liver protein levels and concentrations graphed in GraphPad Prism (San Diego, CA) software.

nCounter analysis

To generate bone marrow derived macrophages (BMDM), bone marrows from the tibia and femora of WT and *Trem2*$^{-/-}$ littermates were flushed out and cultured in DMEM containing 10% FBS and 100ng/ml M-CSF. A homogeneous population of adherent BMDM cells were obtained after 6-days culture. On day 6, the cells were collected and re-

plated for 24hrs. The cells were then treated with 50ng/ml IL-4, and a combination of IL-10 and TGFb for 48h. The cells were subsequently harvested, and RNA was extracted using Direct-zol RNA Miniprep kits (Zymo Research). Comprehensive RNA expression analysis was conducted by nCounter using Myeloid Innate Immunity Panel (NanoString). Extracted total RNA (50ng) was mixed with the master mix and capture ProbeSet in the kit, and the mixture was hybridized for 19 h at 65C. The hybridized samples were immediately loaded into Prep Station, and immobilized RNA samples on the cartridge was analyzed with Digital Analyzer (NanoString). Data analysis was performed using nSolver v4.0 (NanoString).

Flow Cytometry

Non-parenchymal cells (NPC) enriched in monocyte/macrophages were isolated based on previous reports (8-10). Briefly, freshly dissected livers were cut in small pieces and digested in RPMI media containing Collagenase D (2mg/ml) and DNase (0.1-0.2mg/ml) for 20 minutes in orbital shaker (150rpm) at 37C. The content was then press-homogenized through the mesh screen of 100uM Falcon cell strainer with the plunger from a 3-5CC syringe. After centrifugation, the pellet was resuspended in 33% Percoll and centrifuged for 20 min at 500G without brake. After RBC lysis, the pellet was used for flow cytometric analysis. The cells were first incubated with CD16/32 in order to block the Fc receptor, to reduce nonspecific binding. They were then stained with monoclonal antibodies (Table S2) for 30 min to 1 h on ice. The gating strategy for liver macrophages is outlined in Figure S5C-K (as previously reported (11)). Sorting and analysis were performed using a SONY MA900 Cell Sorter (Sony Biotechnology), SONY MA software (v.3.1.1) and FlowJo software (v.10.6.2,BD). The populations of F4/80+Cd9+, F4/80+Clec4f+, and Cd11b+Ly6c+ cells within each mouse group (Foz and Foz Trem2-/- during MASH progression and regression) were quantified as the proportion of live CD45+

cells and were expressed as fold change relative to the Foz+WD 12w (Foz+WD MASH) group.

Biochemical Analyses

Peripheral blood was collected by heart puncture. Plasma was separated using BD microtainer tubes (BD, Franklin Lakes, NJ). The plasma ALT level was measured using the InfinityTM kit (ThermoFischer Scientific, Waltham, MA). Plasma LPS was measured using the LPS-ELISA Kit (Lifeome Biolabs, Inc, Oceanside, CA) and LBP was measured using the LBP-ELISA kit (Abcam, Fremont, CA).

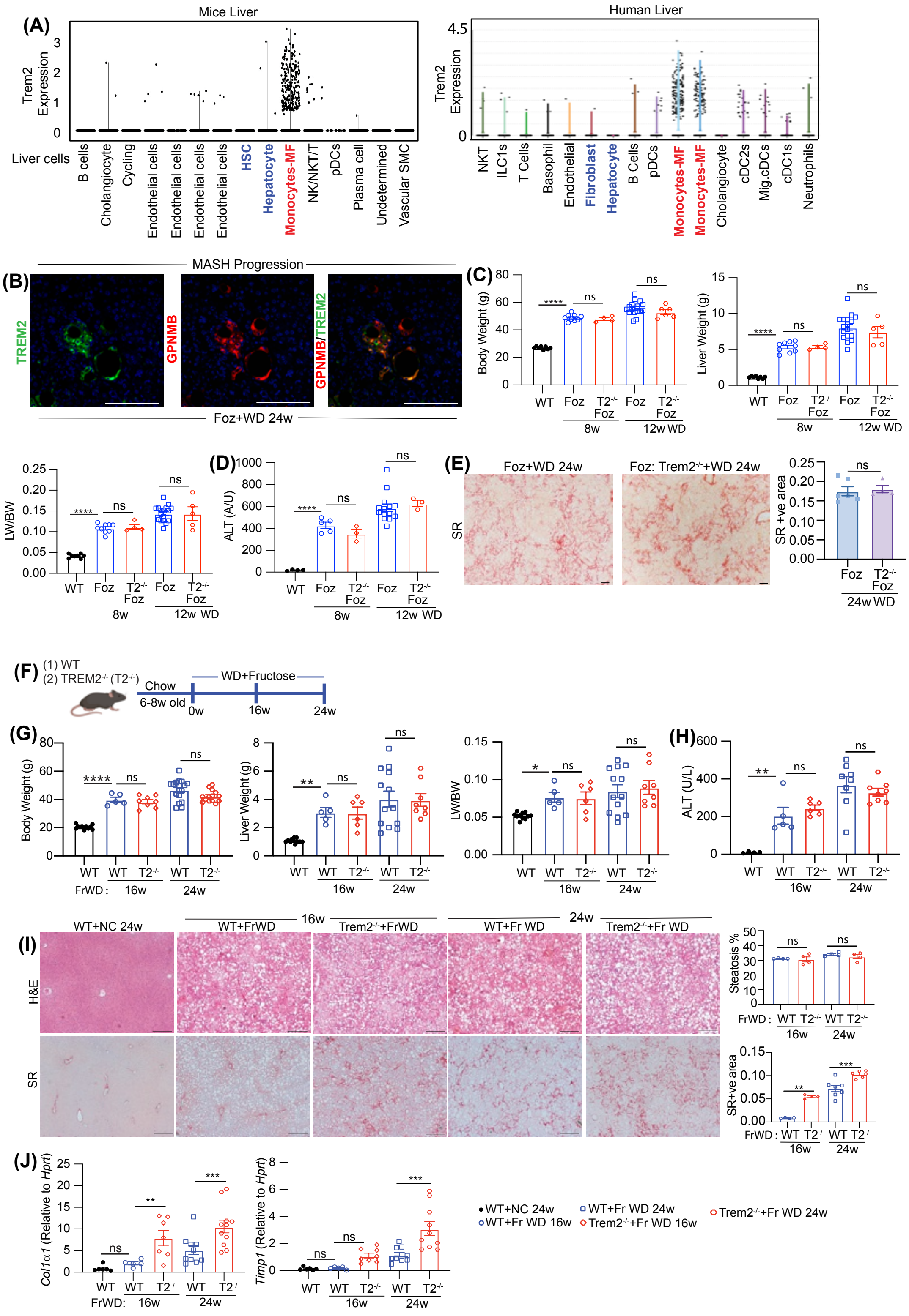
(A)
Mice Liver
Human Liver
Trem2 Expression
Liver cells
B cells
Cholangiocyte
Cycling
Endothelial cells
HSC
Hepatocyte
Monocytes-MF
NK/NKT/T
pDCs
Plasma cell
Undetermined
Vascular SMC
NKT
ILC1s
T Cells
Basophil
Endothelial
Fibroblast
B Cells
Cholangiocyte
cDC2s
Mig.cDCs
cDC1s
Neutrophils
(B)
MASH Progression
TREM2
GPNMB
GPNMB/TREM2
Foz+WD 24w
(C)
Body Weight (g)
Liver Weight (g)
WT
Foz
T2-/- Foz
8w
12w WD
(D)
LW/BW
ALT (A/U)
(E)
Foz+WD 24w
Foz: Trem2-/-+WD 24w
SR
SR +ve area
24w WD
(F)
(1) WT
(2) TREM2-/- (T2-/-)
Chow
6-8w old
WD+Fructose
0w
16w
24w
(G)
(H)
ALT (U/L)
FrWD:
(I)
WT+NC 24w
WT+FrWD
Trem2-/-+FrWD
WT+Fr WD
Trem2-/-+Fr WD
H&E
Steatosis %
SR+ve area
(J)
Col1α1 (Relative to Hprt)
Timp1 (Relative to Hprt)
WT+NC 24w
WT+Fr WD 16w
WT+Fr WD 24w
Trem2-/-+Fr WD 16w
Trem2-/-+Fr WD 24w

Fig. S1.1. TREM2 in MASH. ***(A)*** Violin plot representing the expression levels of *Trem2* in various liver cell types from a proteogenomic atlas of the murine liver (12) (left panel) and an integrated scRNAseq atlas of healthy & diseased human liver of different etiologies (including MASH)(13) (right panel). ***(B)*** FFPE Liver sections from 20w WD fed *Foz/Foz* mice stained with Anti-Trem2 (Left panel) and anti-GPNMB (red) (middle panel) antibodies. (Merged image right panel; DAPI=blue) (Scale Bar 100µm). ***(C-D)*** 6-8 weeks old *Foz/Foz* and *Foz::Trem2*$^{-/-}$ (T2$^{-/-}$ Foz) mice were placed on WD for 8-12 weeks. Changes in ***(C)*** body weight, liver weight, and liver-to-body weight ratio were recorded. ***(D)*** Plasma ALT levels were determined. ***(E)*** Representative SR stained liver sections from 24w WD fed *Foz/Foz* and *Foz::Trem2*$^{-/-}$ mice. Bar graphs show Image J quantifications (Scale Bar 200µm). ***(F)*** 6-8 weeks old WT B6 and *Trem2*$^{-/-}$ (T2$^{-/-}$) mice were placed on a WD along with fructose in drinking water (FrWD) for 16 to 24w. Changes in ***(G)*** body weight, liver weight and liver to body weight ratio were plotted. ***(H)*** Plasma ALT levels were determined. ***(I)*** Representative H&E and SR-stained liver sections and corresponding Image J quantifications (Scale Bar 200µm). ***(J)*** Total liver RNA was subjected to qRT-PCR analysis for *Col1*α*1,* and *Timp1* normalized to *Hprt.* Data is expressed as means±SEM; One-way Anova, T Test; *P<0.05,**P<0.01,***P<0.001,****P<0.0001.

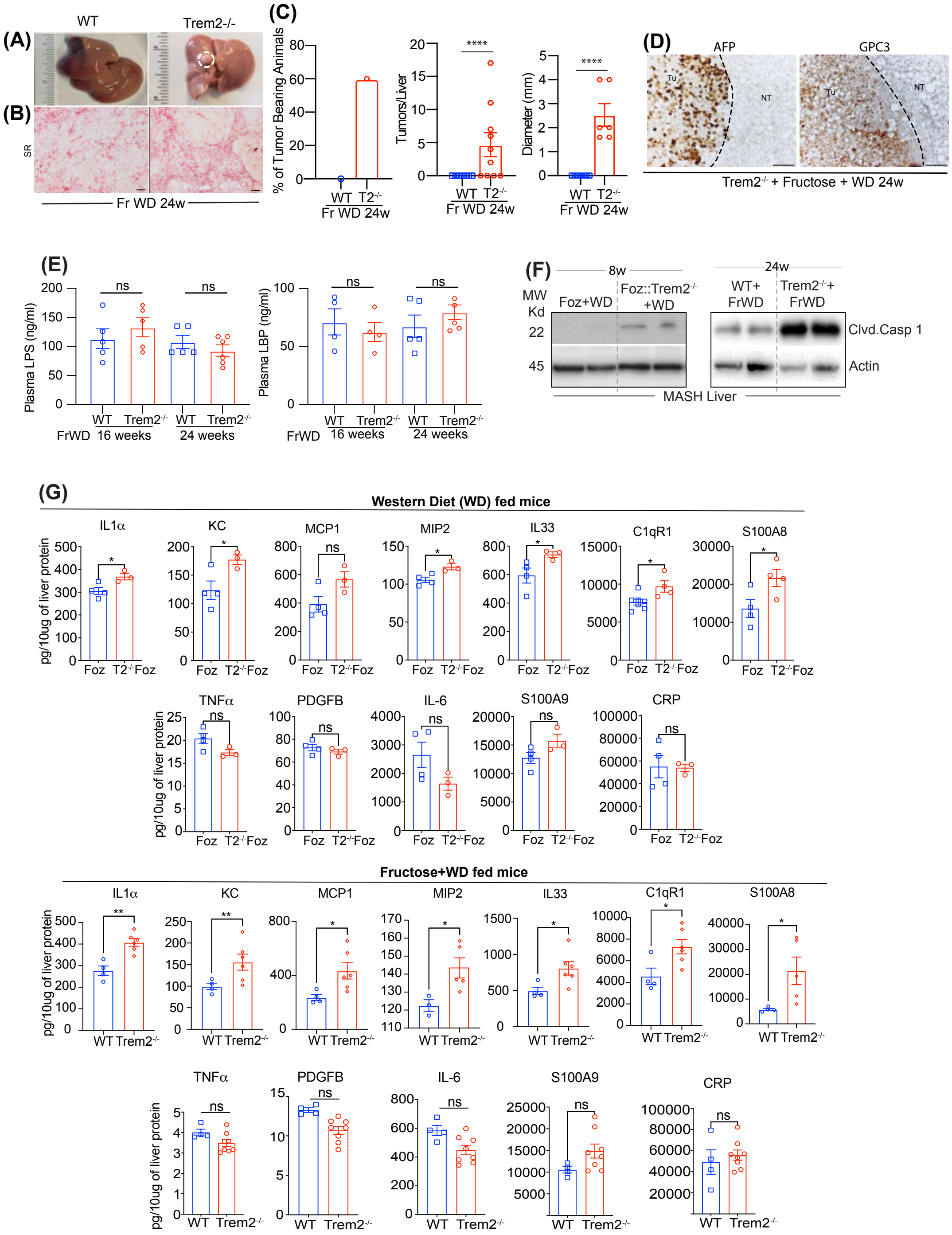
(A)
WT
Trem2-/-
(B)
SR
Fr WD 24w
(C)
% of Tumor Bearing Animals
Tumors/Liver
Diameter (mm)
****
WT T2-/-
Fr WD 24w
(D)
AFP
GPC3
Tu
NT
Trem2-/- + Fructose + WD 24w
(E)
Plasma LPS (ng/ml)
Plasma LBP (ng/ml)
ns
WT Trem2-/- WT Trem2-/-
FrWD 16 weeks 24 weeks
(F)
8w
24w
MW Kd
Foz+WD
Foz::Trem2-/- +WD
WT+ FrWD
Trem2-/-+ FrWD
22
45
Clvd.Casp 1
Actin
MASH Liver
(G)
Western Diet (WD) fed mice
pg/10ug of liver protein
IL1α
KC
MCP1
MIP2
IL33
C1qR1
S100A8
TNFα
PDGFB
IL-6
S100A9
CRP
Foz T2-/-Foz
Fructose+WD fed mice
WT Trem2-/-

Fig. S1.2. **TREM2 in MASH.** ***(A-D)*** WT and *Trem2*$^{-/-}$ mice were fed FrWD for 24w and livers were analyzed. ***(A)*** Representative gross liver images showing macroscopic tumor nodules in *Trem2*$^{-/-}$ mice (white circle) but not in WT littermates. ***(B)*** FFPE liver sections were stained with SR (scale bar 200µm). ***(C)*** Tumor incidence, number of macroscopically visible tumors (>1 mm), and tumor diameters were plotted. ***(D)*** Representative immunohistochemistry (IHC) images showing staining of HCC markers AFP and GPC3 in the tumor region of Trem2$^{-/-}$ mice (scale bar 200µm). ***(E)*** FrWD-fed WT and Trem2$^{-/-}$ mice were sacrificed at the indicated time points and plasma LPS and LBP levels were determined using ELISA. ***(F)*** Liver lysates from the indicated mice were subjected to immunoblot analysis to identify the extent of Caspase 1 cleavage. ***(G)*** Liver lysates from 8w WD fed *Foz/Foz* and *Foz::Trem2*$^{-/-}$ (T2$^{-/-}$ Foz) (upper panels) and 16w FrWD fed WT and *Trem2*$^{-/-}$ mice (lower panels) were subjected to Luminex analyses to quantify differences in the levels of indicated cytokines and chemokines. Data is expressed as means±SEM; One-way Anova, T Test; *$P<0.05$,**$P<0.01$,****$P<0.0001$.

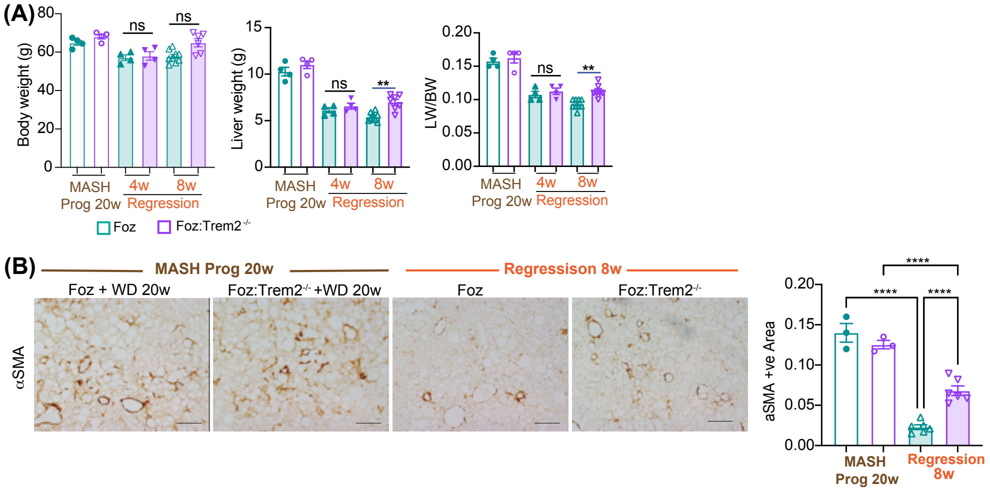

Fig. S2. Absence of TREM2 prevents effective MASH and fibrosis resolution. 12w WD-fed *Foz/Foz* and *Foz::Trem2*$^{-/-}$ mice, were either switched to a chow diet for an additional 4-8w to model MASH regression or continued on WD as age-matched controls (MASH progression 20w) ***(A)*** Changes in body weight, liver weight, and liver-to-body weight ratio were plotted. ***(B)*** FFPE liver sections from indicated mice were subjected to αSMA staining (Scale Bar 100µm). The tissue staining was quantified by ImageJ and plotted. Data is expressed as means±SEM; One-way Anova, **P<0.01,****P<0.0001.

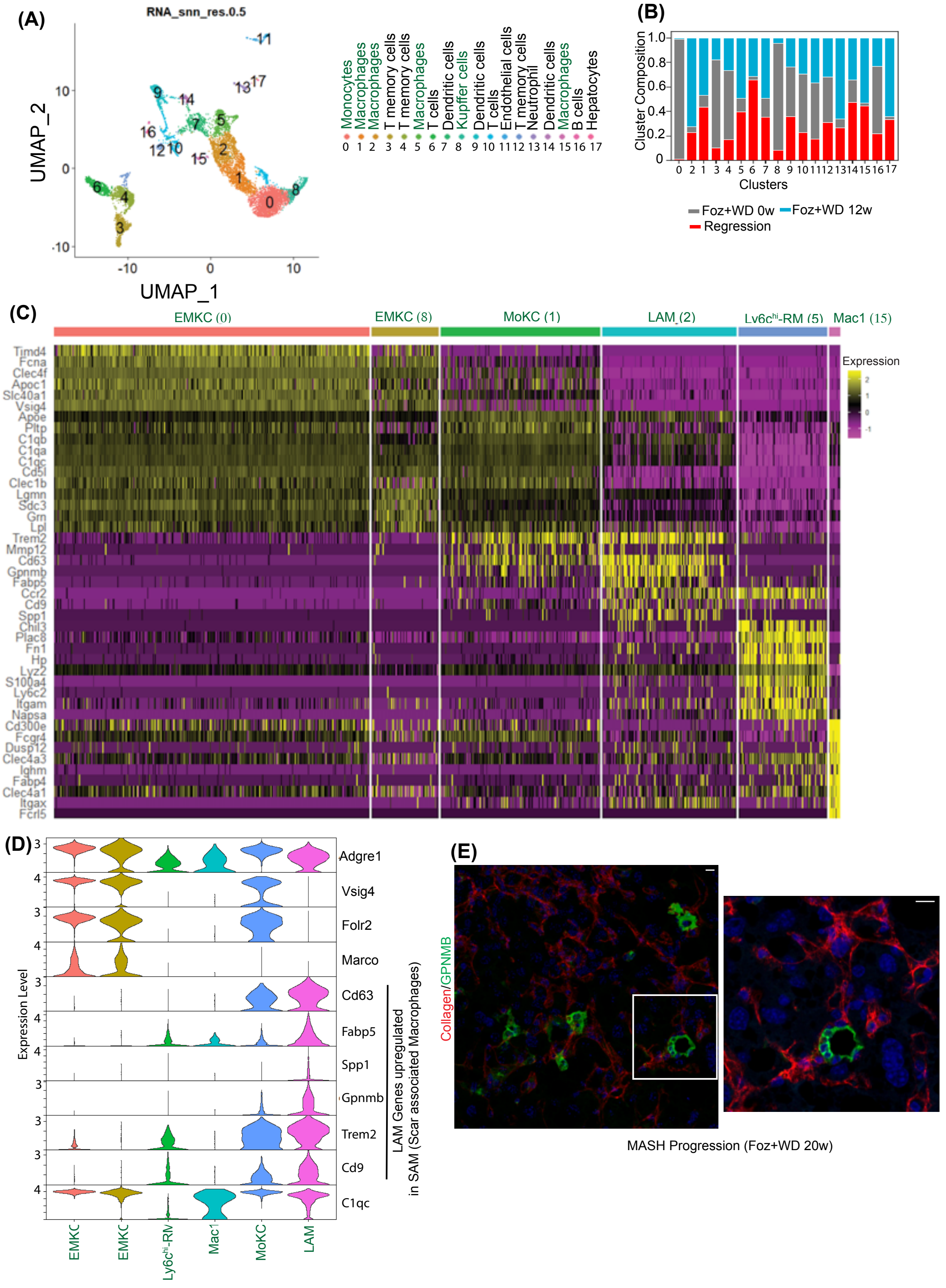

(A)
RNA_snn_res.0.5
UMAP_1
UMAP_2
0 Monocytes
1 Macrophages
2 Macrophages
3 T memory cells
4 T memory cells
5 Macrophages
6 T cells
7 Dendritic cells
8 Kupffer cells
9 Dendritic cells
10 T cells
11 Endothelial cells
12 T memory cells
13 Neutrophil
14 Dendritic cells
15 Macrophages
16 B cells
17 Hepatocytes
(B)
Cluster Composition
Clusters
Foz+WD 0w
Foz+WD 12w
Regression
(C)
EMKC (0)
EMKC (8)
MoKC (1)
LAM (2)
Ly6c^hi-RM (5)
Mac1 (15)
Expression
Timd4
Fcna
Clec4f
Apoc1
Slc40a1
Vsig4
Apoe
Pltp
C1qb
C1qa
C1qc
Cd5l
Clec1b
Lgmn
Sdc3
Grn
Lpl
Trem2
Mmp12
Cd63
Gpnmb
Fabp5
Ccr2
Cd9
Spp1
Chil3
Plac8
Fn1
Hp
Lyz2
S100a4
Ly6c2
Itgam
Napsa
Cd300e
Fcgr4
Dusp12
Clec4a3
Ighm
Fabp4
Clec4a1
Itgax
Fcrl5
(D)
Expression Level
Adgre1
Vsig4
Folr2
Marco
Cd63
Fabp5
Spp1
Gpnmb
Trem2
Cd9
C1qc
LAM Genes upregulated in SAM (Scar associated Macrophages)
EMKC
EMKC
Ly6c^hi-RM
Mac1
MoKC
LAM
(E)
Collagen/GPNMB
MASH Progression (Foz+WD 20w)

Fig. S3. Macrophage heterogeneity during MASH progression and regression. ***(A)*** UMAP showing 18 clusters identified from scRNAseq analysis of immune-enriched cells isolated from healthy control (Foz+WD 0w), MASH (Foz+WD 12w) and regression mice. ***(B)*** Cluster composition plot showing the relative proportion of cells from healthy, MASH and regression livers across all the clusters. ***(C)*** Heatmap of key differentially expressed genes in each cell across all monocyte/macrophage clusters. Cluster identity is established from these differentially expressed genes based on previous reports (14-18) and are represented on the top with the cluster number in parenthesis. EMKC: embryo-derived Kupffer Cells, MoKC: Monocyte-derived Kupffer cells, LAM: Lipid associated macrophages, Ly6c^{hi}-RM: Ly6c high recruited macrophage, Mac1: macrophage cluster highly expressing Itgax, Clec4a1. ***(D)*** Violin plot showing the expression of human SAM marker genes (Cd63+Fabp5+Spp1+Gpnmb+Trem2+Cd9+) across all the 6 monocyte/macrophage clusters in *Foz/Foz* mice, indicating overlapping gene signatures in LAM and SAM. ***(E)*** FFPE liver sections from 20w WD fed Foz mice were co-stained with anti-Type 1 Collagen (red) and anti-GPNMB (green) antibodies (Scale Bar=10μm).

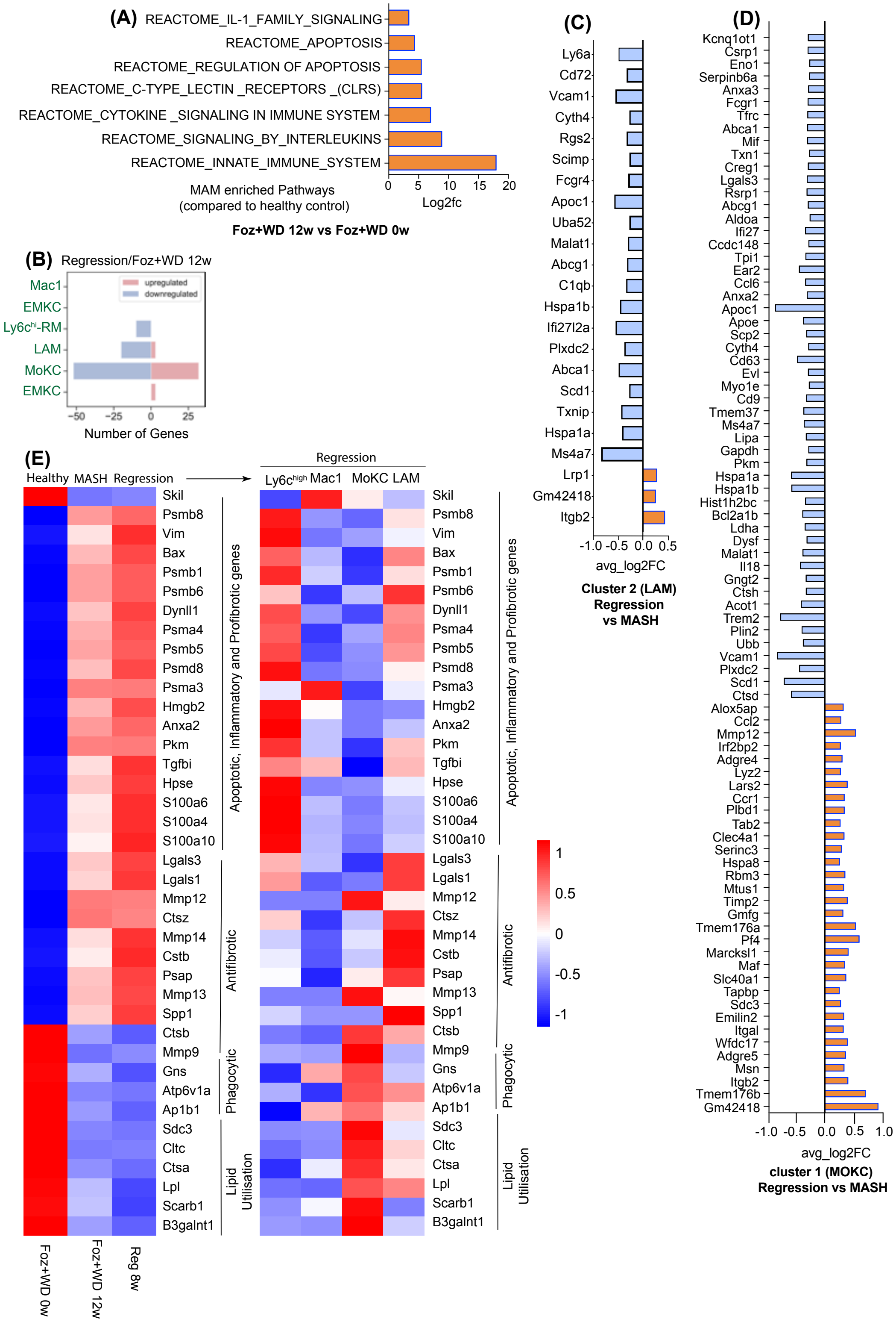
(A)
REACTOME_IL-1_FAMILY_SIGNALING
REACTOME_APOPTOSIS
REACTOME_REGULATION OF APOPTOSIS
REACTOME_C-TYPE_LECTIN _RECEPTORS _(CLRS)
REACTOME_CYTOKINE _SIGNALING IN IMMUNE SYSTEM
REACTOME_SIGNALING_BY_INTERLEUKINS
REACTOME_INNATE_IMMUNE_SYSTEM
0 5 10 15 20
Log2fc
MAM enriched Pathways
(compared to healthy control)
Foz+WD 12w vs Foz+WD 0w
(B) Regression/Foz+WD 12w
upregulated
downregulated
Mac1
EMKC
Ly6chi-RM
LAM
MoKC
EMKC
−50 −25 0 25
Number of Genes
(C)
Ly6a
Cd72
Vcam1
Cyth4
Rgs2
Scimp
Fcgr4
Apoc1
Uba52
Malat1
Abcg1
C1qb
Hspa1b
Ifi27l2a
Plxdc2
Abca1
Scd1
Txnip
Hspa1a
Ms4a7
Lrp1
Gm42418
Itgb2
-1.0 -0.5 0.0 0.5
avg_log2FC
Cluster 2 (LAM)
Regression
vs MASH
(D)
Kcnq1ot1
Csrp1
Eno1
Serpinb6a
Anxa3
Fcgr1
Tfrc
Abca1
Mif
Txn1
Creg1
Lgals3
Rsrp1
Abcg1
Aldoa
Ifi27
Ccdc148
Tpi1
Ear2
Ccl6
Anxa2
Apoc1
Apoe
Scp2
Cyth4
Cd63
Evl
Myo1e
Cd9
Tmem37
Ms4a7
Lipa
Gapdh
Pkm
Hspa1a
Hspa1b
Hist1h2bc
Bcl2a1b
Ldha
Dysf
Malat1
Il18
Gngt2
Ctsh
Acot1
Trem2
Plin2
Ubb
Vcam1
Plxdc2
Scd1
Ctsd
Alox5ap
Ccl2
Mmp12
Irf2bp2
Adgre4
Lyz2
Lars2
Ccr1
Plbd1
Tab2
Clec4a1
Serinc3
Hspa8
Rbm3
Mtus1
Timp2
Gmfg
Tmem176a
Pf4
Marcksl1
Maf
Slc40a1
Tapbp
Sdc3
Emilin2
Itgal
Wfdc17
Adgre5
Msn
Itgb2
Tmem176b
Gm42418
-1.0 -0.5 0.0 0.5 1.0
avg_log2FC
cluster 1 (MOKC)
Regression vs MASH
(E)
Healthy MASH Regression
Regression
Ly6chigh Mac1 MoKC LAM
Skil
Psmb8
Vim
Bax
Psmb1
Psmb6
Dynll1
Psma4
Psmb5
Psmd8
Psma3
Hmgb2
Anxa2
Pkm
Tgfbi
Hpse
S100a6
S100a4
S100a10
Lgals3
Lgals1
Mmp12
Ctsz
Mmp14
Cstb
Psap
Mmp13
Spp1
Ctsb
Mmp9
Gns
Atp6v1a
Ap1b1
Sdc3
Cltc
Ctsa
Lpl
Scarb1
B3galnt1
Apoptotic, Inflammatory and Profibrotic genes
Antifibrotic
Phagocytic
Lipid Utilisation
1
0.5
0
-0.5
-1
Foz+WD 0w
Foz+WD 12w
Reg 8w

Fig. S4. Changes in gene signature of various macrophage sub-population during MASH and regression. ***(A)*** Pathway enrichment analysis of all macrophage clusters (clusters 0, 1, 2, 5, 8 and 15) from the scRNAseq data showing selected significantly differentially expressed pro-inflammatory pathways in macrophages from MASH livers compared to macrophage from healthy controls, represented as a bar plot. ***(B)*** Bar plot depicting the number of genes upregulated (red) or downregulated (blue) in each macrophage cluster during regression compared to MASH progression. ***(C-D)*** All the genes upregulated and downregulated during MASH regression compared to MASH progression in cluster 2 (LAM) ***(C)*** and cluster 1 (MoKC) ***(D)***, are represented as a bar plot. ***(E)*** Heat map (left) showing the relative expression levels of the indicated genes across healthy (Foz+WD 0w), MASH progression (Foz+WD 12w), and MASH regression (Reg 8w) groups. First, all the genes that are significantly ($p<0.05$) altered in MASH vs healthy were determined, subsequently the expression of these genes during MASH regression was plotted as the heatmap. Key genes with established function in their corresponding pathways as indicated on the right are plotted. The heat map on the right shows the expression profiles of these genes across various macrophage sub-types during regression.

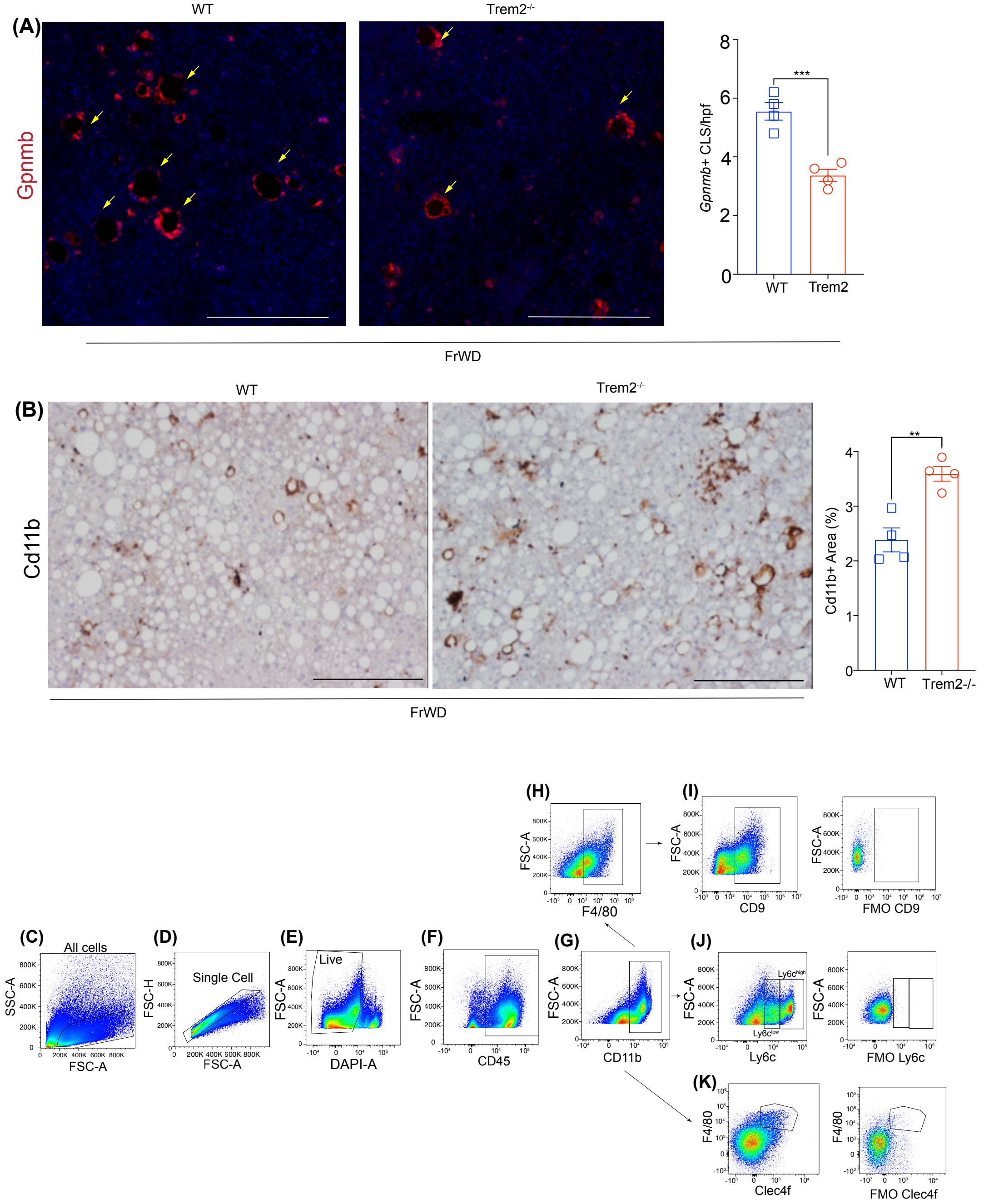
(A)
WT
Trem2-/-
Gpnmb
Gpnmb+ CLS/hpf
***
WT
Trem2
FrWD
(B)
WT
Trem2-/-
Cd11b
Cd11b+ Area (%)
**
WT
Trem2-/-
FrWD
(C)
All cells
SSC-A
FSC-A
(D)
Single Cell
FSC-H
FSC-A
(E)
Live
FSC-A
DAPI-A
(F)
FSC-A
CD45
(G)
FSC-A
CD11b
(H)
FSC-A
F4/80
(I)
FSC-A
CD9
FSC-A
FMO CD9
(J)
Ly6chigh
Ly6clow
FSC-A
Ly6c
FSC-A
FMO Ly6c
(K)
F4/80
Clec4f
F4/80
FMO Clec4f

Fig. S5. Absence of *Trem2* suppresses emergence of LAMs during MASH progression and regression. WT and *Trem2*$^{-/-}$ mice were fed FrWD for 24w and livers were analyzed. ***(A)*** FFPE liver sections were stained with anti-GPNMB (red) antibody and DAPI (blue) (scale Bar 200μm) with quantification showing the number of GPNMB^{+} LAM enriched hCLS in each randomly selected high-power field (hpf). ***(B)*** Cd11b stained FFPE liver sections from indicated mice (scale Bar 200μm) with bar plot showing ImageJ quantifications. ***(C-K)*** Flow cytometric analysis of liver non-parenchymal cells isolated from Foz and *Foz::Trem2*$^{-/-}$ mice undergoing MASH progression and regression for CD9^{+}, Clec4f^{+} and Ly6C^{hi} macrophage subpopulations. NPC were isolated by 33% percoll method that enriches the monocyte/macrophage population (10). ***(C)*** Forward and side scatter gating on lymphocytes to exclude cellular debris, ***(D)*** Singlets gating to exclude doublets, ***(E)*** Live cell positive gate, Gating on ***(F)*** CD45^{+} cells ***(G)*** CD11b^{+} cells and ***(H)*** F4/80^{+} cells. Subsequently from the F4/80^{+} cells we gated for ***(I)*** CD9^{+} cells indicating Cluster 2 (representing primarily LAM). Further from the Cd11b population, we gated for Ly6c^{low} and Ly6c^{hi} cells. The Ly6c^{hi} subpopulation ***(J)*** indicates cluster 5 of scRNAseq. Finally, from the Cd11b gate (G) we performed F4/80^{+} and Clec4f^{+} positive gating indicating MoKC ***(K)***. Notably, even though Clef4f is also expressed in clusters 0 and 8, both these clusters almost exclusively represent macrophages from healthy mice (Figure 3B). Therefore, Clec4f^{+} macrophages derived from MASH and regression mice indicate MoKC. The corresponding FMO (full stained minus one) of the final gates are indicated on the right. Data is expressed as means±SEM; T Test; **P<0.01,***P<0.001.

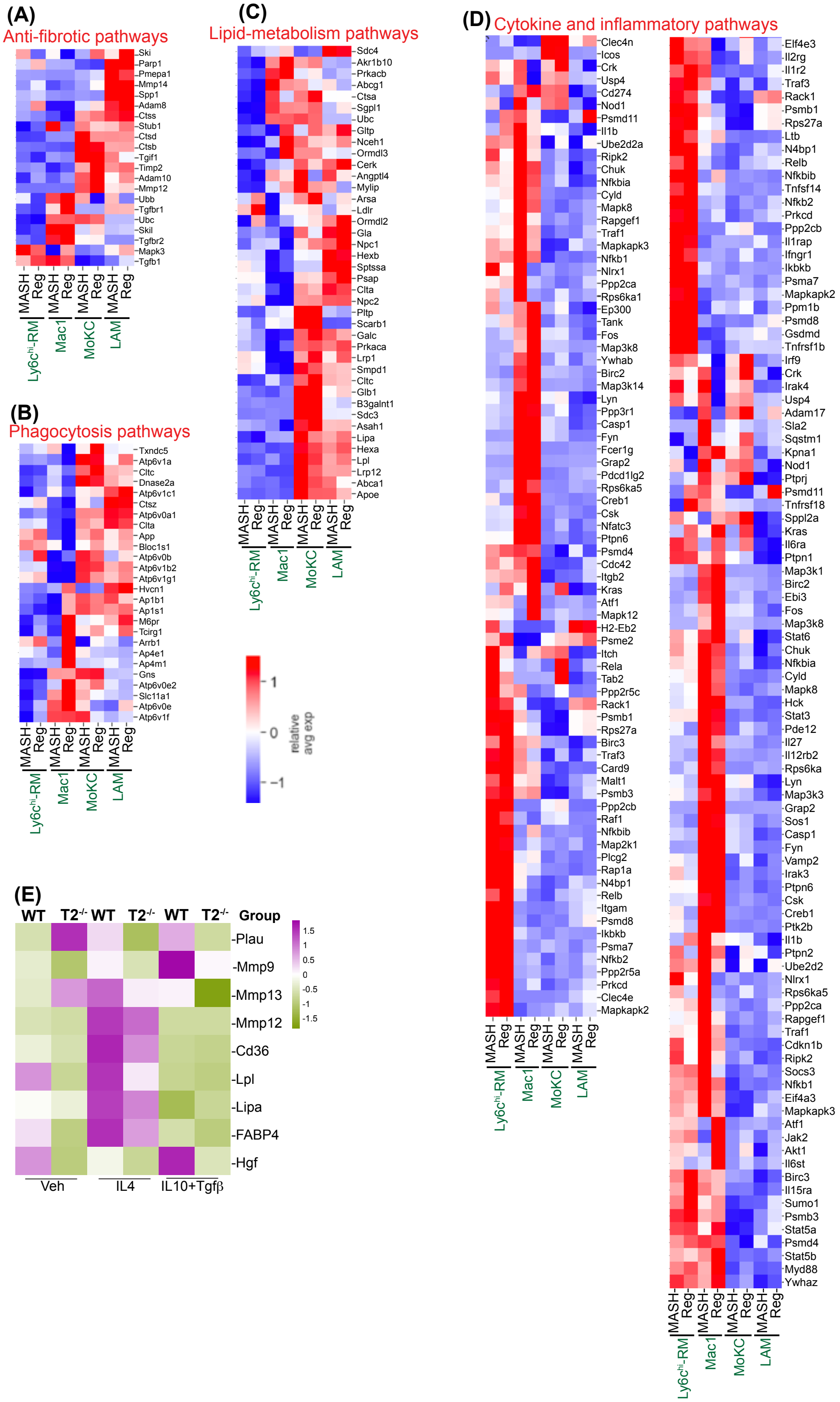
(A)
Anti-fibrotic pathways
Ski
Parp1
Pmepa1
Mmp14
Spp1
Adam8
Ctss
Stub1
Ctsd
Ctsb
Tgif1
Timp2
Adam10
Mmp12
Ubb
Tgfbr1
Ubc
Skil
Tgfbr2
Mapk3
Tgfb1
MASH
Reg
Ly6c^hi-RM
Mac1
MoKC
LAM
(B)
Phagocytosis pathways
Txndc5
Atp6v1a
Cltc
Dnase2a
Atp6v1c1
Ctsz
Atp6v0a1
Clta
App
Bloc1s1
Atp6v0b
Atp6v1b2
Atp6v1g1
Hvcn1
Ap1b1
Ap1s1
M6pr
Tcirg1
Arrb1
Ap4e1
Ap4m1
Gns
Atp6v0e2
Slc11a1
Atp6v0e
Atp6v1f
(C)
Lipid-metabolism pathways
Sdc4
Akr1b10
Prkacb
Abcg1
Ctsa
Sgpl1
Ubc
Gltp
Nceh1
Ormdl3
Cerk
Angptl4
Mylip
Arsa
Ldlr
Ormdl2
Gla
Npc1
Hexb
Sptssa
Psap
Clta
Npc2
Pltp
Scarb1
Galc
Prkaca
Lrp1
Smpd1
Cltc
Glb1
B3galnt1
Sdc3
Asah1
Lipa
Hexa
Lpl
Lrp12
Abca1
Apoe
1
0
-1
relative avg exp
(D) Cytokine and inflammatory pathways
Clec4n
Icos
Crk
Usp4
Cd274
Nod1
Psmd11
Il1b
Ube2d2a
Ripk2
Chuk
Nfkbia
Cyld
Mapk8
Rapgef1
Traf1
Mapkapk3
Nfkb1
Nlrx1
Ppp2ca
Rps6ka1
Ep300
Tank
Fos
Map3k8
Ywhab
Birc2
Map3k14
Lyn
Ppp3r1
Casp1
Fyn
Fcer1g
Grap2
Pdcd1lg2
Rps6ka5
Creb1
Csk
Nfatc3
Ptpn6
Psmd4
Cdc42
Itgb2
Kras
Atf1
Mapk12
H2-Eb2
Psme2
Itch
Rela
Tab2
Ppp2r5c
Rack1
Psmb1
Rps27a
Birc3
Traf3
Card9
Malt1
Psmb3
Ppp2cb
Raf1
Nfkbib
Map2k1
Plcg2
Rap1a
N4bp1
Relb
Itgam
Psmd8
Ikbkb
Psma7
Nfkb2
Ppp2r5a
Prkcd
Clec4e
Mapkapk2
Elf4e3
Il2rg
Il1r2
Traf3
Rack1
Psmb1
Rps27a
Ltb
N4bp1
Relb
Nfkbib
Tnfsf14
Nfkb2
Prkcd
Ppp2cb
Il1rap
Ifngr1
Ikbkb
Psma7
Mapkapk2
Ppm1b
Psmd8
Gsdmd
Tnfrsf1b
Irf9
Crk
Irak4
Usp4
Adam17
Sla2
Sqstm1
Kpna1
Nod1
Ptprj
Psmd11
Tnfrsf18
Sppl2a
Kras
Il6ra
Ptpn1
Map3k1
Birc2
Ebi3
Fos
Map3k8
Stat6
Chuk
Nfkbia
Cyld
Mapk8
Hck
Stat3
Pde12
Il27
Il12rb2
Rps6ka
Lyn
Map3k3
Grap2
Sos1
Casp1
Fyn
Vamp2
Irak3
Ptpn6
Csk
Creb1
Ptk2b
Il1b
Ptpn2
Ube2d2
Nlrx1
Rps6ka5
Ppp2ca
Rapgef1
Traf1
Cdkn1b
Ripk2
Socs3
Nfkb1
Eif4a3
Mapkapk3
Atf1
Jak2
Akt1
Il6st
Birc3
Il15ra
Sumo1
Psmb3
Stat5a
Psmd4
Stat5b
Myd88
Ywhaz
(E)
WT
T2-/-
Group
Plau
Mmp9
Mmp13
Mmp12
Cd36
Lpl
Lipa
FABP4
Hgf
1.5
1
0.5
0
-0.5
-1
-1.5
Veh
IL4
IL10+Tgfβ

Fig. S6. Comparison of gene signatures in various macrophage sub-populations in MASH vs regression. ***(A-D)*** scRNAseq data from MASH and regression macrophage sub-populations (clusters 1, 2, 5 and 15) was analyzed as described in Figures 3 and 4. *Trem2*-correlated genes that are significantly altered are represented as heat maps showing relative expression among the various macrophage sub-populations during MASH vs regression (Reg). The genes in the heat maps were categorized based on the following meta pathways: ***(A)*** Anti-fibrotic, ***(B)*** Phagocytosis, ***(C)*** Lipid metabolism, ***(D)*** Cytokine and inflammatory. ***(E)*** nCounter analysis. BMDMs isolated from WT and *Trem2*$^{-/-}$ mice were polarized with IL-4, and IL-10+TGFβ for 48h (M2a and M2c). RNA expression analysis was conducted by nCounter using Myeloid Innate Immunity Panel (NanoString). Heat-map showing relative expression of indicated genes belonging to reparative pathways.

Table S1. List of primers for qRT-PCR

Gene	Sequence (5'->3')	
	Forward primer	Reverse primer
Col1α1	TAGGCCATTGTGTATGCAGC	ACATGTTCAGCTTTGTGGACC
Timp1	AGGTGGTCTCGTTGATTTCT	GTAAGGCCTGTAGCTGTGCC
Trem2	CTGGAACCGTCACCATCACTC	CGAAACTCGATGACTCCTCGG
Mcp1	ATTGGGATCATCTTGCTGGT	CCTGCTGTTCACAGTTGCC
TyroBP	CCCAAGATGCGACTGTTCTTC	GTCCCTTGACCTCGGGAGA
Cd11b	GTTTGTTGAAGGCATTTCCC	ATTCGGTGATCCCTTGGATT
Col3a1	TAGGACTGACCAAGGTGGCT	GGAACCTGGTTTCTTCTCACC
Afp	ACAGGAGGCTATGCATCACC	TGGACATCTTCACCATGTGG
Gpc3	CCCTGAATCTCGGAATTGAA	AGTCCCTGGCAGTAAGAGCA
Golm1	CGTCGCAGCATGAAGTCTC	CAGTAGTTGAAGCCTAGCACAAT
Hprt	GTTAAGCAGTACAGCCCCAAA	AGGGCATATCCAACAACAAACTT
Il1b	TTGTTGATGTGCTGCTGTGA	TGTGAAATGCCACCTTTT
Il1a	TGAGTTTTGGTGTTTCTGGC	ATGTATGCCTACTCGTCGGG
Gpnmb	GCTGGTCTTCGGATGAAAATGA	CCACAAAGGTGATATTGGAACCC

List of human primers for RT-QPCR

Gene	Sequence (5'->3')	
	Forward primer	Reverse primer
TREM2	GGTCAGCACGCACAACTTG	CGCAGCGTAATGGTGAGAGT
TyroBP	ACTGAGACCGAGTCGCCTTAT	ATACGGCCTCTGTGTGTTGAG
HPRT	CCTGGCGTCGTGAATAGTGAT	AGACGTTCAGTCCTGTCCATAA

Table S2. List of Antibodies

Immunohistochemistry		
Antibody	**Catalog**	**Vendor**
Anti-Collagen I	ab138492	Abcam
Anti-Collagen I	ab279711	Abcam
αSMA	ab5694	Abcam
Trem2	mabn755	Millipore-Sigma
GPC3	251482	Abbiotec
AFP	AF5369-SP	Novus Biologicals
GPNMB	cs90205S	Cell Signaling
Cd11b	ab133357	Abcam
**Immunoblot**		
Antibody	**Catalog**	**Vendor**
Cleaved Caspase 1	NB100-56565ss	Novus Biologicals
Beta-actin	A5441	Millipore-Sigma
Desmin	RB-9014-P0	Thermo Fisher
αSMA	ab5694	Abcam
**Flow Cytometry**		
Antibody	**Catalog**	**Vendor**
Anti-mouse CD45 (30-F11) PerCP-Cy5.5	Biolegend	Cat#103131; RRID: AB_893344
Anti-mouse CD11b (M1/70) BV510	Biolegend	Cat#101245; RRID:AB_2561390
Anti-mouse Ly6C (HK 1.4) BV785	Biolegend	Cat#128041; RRID:AB_2565852
Anti-mouse F4/80 (BM8) PE	Biolegend	Cat#123110; RRID:AB_893486
Anti-mouse CD9 (MZ3) PE-Dazzle	Biolegend	Cat#124822; PRID:AB_2800602
Anti-human/mouse Trem2 A488	R&D	Cat#FAB17291G; PRID:AB_88452
Anti-mouse Siglec F (E50-2440) BV605	BD Biosciences	Cat#740388; RRID:AB_2740118
Anti-mouse Clec4F (3E3F9) A647	Biolegend	Cat#156804, RRID:AB_2814082
Anti-mouse CD16/CD32	Biolegend	Cat#101330; RRID:AB_2561482

Legends for Datasets S1 to S3

Dataset S1 (Separate file). Single cell RNAseq of Liver Non-Parenchymal cells (NPC) - Cluster Identity based on Panglao database

Dataset S2 (Separate file). Trem2+ve and Trem2-ve correlated meta-pathways and individual pathways during MASH progression. Each meta-pathway is depicted in individual tabs. Only significant pathways with FDR<0.3 are shown.

Dataset S3 (Separate file). Trem2+ve and Trem2-ve correlated meta-pathways and individual pathways during MASH regression. Each meta-pathway is depicted in individual tabs. Only significant pathways with FDR<0.3 are shown.

SI REFERENCES

1. N. Renier *et al.*, iDISCO: a simple, rapid method to immunolabel large tissue samples for volume imaging. *Cell* **159**, 896-910 (2014).
2. S. B. Rosenthal *et al.*, Heterogeneity of hepatic stellate cells in a mouse model of non-alcoholic steatohepatitis (NASH). *Hepatology* 10.1002/hep.31743 (2021).
3. S. A. Fleit, H. B. Fleit, S. Zolla-Pazner, Culture and recovery of macrophages and cell lines from tissue culture-treated and -untreated plastic dishes. *J Immunol Methods* **68**, 119-129 (1984).
4. T. Okada *et al.*, Liver Resident Macrophages (Kupffer Cells) Share Several Functional Antigens in Common with Endothelial Cells. *Scand J Immunol* **83**, 139-150 (2016).
5. E. Pfeiffer *et al.*, Featured Article: Isolation, characterization, and cultivation of human hepatocytes and non-parenchymal liver cells. *Exp Biol Med (Maywood)* **240**, 645-656 (2015).
6. A. Subramanian *et al.*, Gene set enrichment analysis: a knowledge-based approach for interpreting genome-wide expression profiles. *Proc Natl Acad Sci U S A* **102**, 15545-15550 (2005).
7. S. Ganguly *et al.*, Nonalcoholic Steatohepatitis and HCC in a Hyperphagic Mouse Accelerated by Western Diet. *Cell Mol Gastroenterol Hepatol* **12**, 891-920 (2021).
8. S. Daemen, M. M. Chan, J. D. Schilling, Comprehensive analysis of liver macrophage composition by flow cytometry and immunofluorescence in murine NASH. *STAR Protoc* **2**, 100511 (2021).
9. R. W. Lynch *et al.*, An efficient method to isolate Kupffer cells eliminating endothelial cell contamination and selective bias. *J Leukoc Biol* **104**, 579-586 (2018).
10. M. E. Moreno-Fernandez, M. Damen, S. Divanovic, A protocol for isolation of primary human immune cells from the liver and mesenteric white adipose tissue biopsies. *STAR Protoc* **2**, 100937 (2021).
11. T. V. Rohm *et al.*, Adipose tissue macrophages secrete small extracellular vesicles that mediate rosiglitazone-induced insulin sensitization. *Nat Metab* **6**, 880-898 (2024).
12. M. Guilliams *et al.*, Spatial proteogenomics reveals distinct and evolutionarily conserved hepatic macrophage niches. *Cell* **185**, 379-396 e338 (2022).
13. J. Brancale, S. Vilarinho, A single cell gene expression atlas of 28 human livers. *J Hepatol* **75**, 219-220 (2021).
14. S. Daemen *et al.*, Dynamic Shifts in the Composition of Resident and Recruited Macrophages Influence Tissue Remodeling in NASH. *Cell Rep* **34**, 108626 (2021).
15. P. Ramachandran *et al.*, Resolving the fibrotic niche of human liver cirrhosis at single-cell level. *Nature* 10.1038/s41586-019-1631-3 (2019).
16. J. S. Seidman *et al.*, Niche-Specific Reprogramming of Epigenetic Landscapes Drives Myeloid Cell Diversity in Nonalcoholic Steatohepatitis. *Immunity* **52**, 1057-1074 e1057 (2020).
17. X. Xiong *et al.*, Landscape of Intercellular Crosstalk in Healthy and NASH Liver Revealed by Single-Cell Secretome Gene Analysis. *Molecular cell* **75**, 644-660 e645 (2019).

18. A. Remmerie *et al.*, Osteopontin Expression Identifies a Subset of Recruited Macrophages Distinct from Kupffer Cells in the Fatty Liver. *Immunity* **53**, 641-657 e614 (2020).
